# Harnessing lipid-driven immunometabolic pathways in omental metastases to enhance immunotherapy in patients with ovarian cancer

**DOI:** 10.1038/s41392-026-02594-8

**Published:** 2026-03-04

**Authors:** Meggy Suarez-Carmona, Mareike Hampel, Xin-Wen Zhang, Alexandra Pöchmann, Silke A. Grauling-Halama, Nektarios A. Valous, Pornpimol Charoentong, Dyke Ferber, Jannis Wissfeld, Alicia Höflich, Stanislas Goriely, Aurélie Detavernier, Abdulkader Azouz, Anthony Rongvaux, Sven Zukunft, Ingrid Fleming, Jürgen G. Okun, Vickie Baracos, Mathias Heikenwalder, Laurence Zitvogel, Xinyi Xu, Chenqi Xu, Michael Volkmar, Daniel Schraivogel, Lars Steinmetz, Junzo Hamanishi, Masaki Mandai, Matthias Gaida, Theresa Mokry, Johanna Nattenmüller, Oliver Sedlaczek, Nanna Monje, Roxana Schwab, Annette Hasenburg, Athanasios Mavratzas, Regina Johanna Boger, Frederik Marmé, Sarah Schott, Niels Halama

**Affiliations:** 1 German Cancer Research Center (DKFZ), Heidelberg, Germany; 2 National Center for Tumor Diseases, Department of Medical Oncology, University Hospital Heidelberg, Heidelberg, Germany; 3 Department of Tumor Immunology and Tumor Immunotherapy, Helmholtz Center for Translational Oncology (HI-TRON), Mainz, Germany; 4 Faculty of Biosciences (AP) and Faculty of Medicine (NH), Heidelberg University, Heidelberg, Germany; 5 Applied Tumor Immunity Clinical Cooperation Unit, National Center for Tumor Diseases (NCT), German Cancer Research Center, Heidelberg, Germany; 6 Center for Quantitative Analysis of Molecular and Cellular Biosystems (Bioquant), University Heidelberg, Heidelberg, Germany; 7 Department of mRNA Cancer Immunotherapies, Helmholtz Center for Translational Oncology (HI-TRON), Mainz, Germany; 8 Institute of Medical Immunology (IMI) and Immunobiology Lab, ULB Center for Research in Immunology (U-CRI), Gosselies, Belgium; 9 Translational Science and Therapeutics Division, Fred Hutchinson Cancer Center, Seattle, WA USA; 10 Institute for Vascular Signaling, Centre for Molecular Medicine, Goethe University, Frankfurt, Germany; 11 German Center of Cardiovascular Research (DZHK), Partner site Rhein-Main, Frankfurt am Main, Germany; 12 Dietmar Hopp Metabolic Center, University Children’s Hospital Heidelberg, Heidelberg, Germany; 13 Division of Palliative Care Medicine, Department of Oncology, University of Alberta, Edmonton, AB Canada; 14 German Cancer Research Center (DKFZ), Division of Chronic inflammation and cancer, Heidelberg, Germany; 15 Institut Gustave-Roussy, Paris, France; 16 State Key Laboratory of Molecular Biology, Shanghai Science Research Center, CAS Center for Excellence in Molecular Cell Science, Shanghai Institute of Biochemistry and Cell Biology, Chinese Academy of Sciences, University of Chinese Academy of Sciences, Shanghai, China; 17 T Cell Discovery Platform, Helmholtz Institute for Translational Oncology (HI-TRON) Mainz – A Helmholtz Institute of the DKFZ, Mainz, Germany; 18 Genome Biology Unit, European Molecular Biology Laboratory (EMBL), Heidelberg, Germany; 19 Department of Genetics, Stanford University School of Medicine, Stanford, CA USA; 20 Stanford Genome Technology Center, Palo Alto, CA USA; 21 Department of Gynecology and Obstetrics, Kyoto University Graduate School of Medicine, Kyoto, Japan; 22 Institute of Pathology, University Medical Center Mainz, Mainz, Germany; 23 TRON, Translational Oncology at the University Medical Center, JGU-Mainz, Mainz, Germany; 24 Department of Diagnostic and Interventional Radiology, Clinic of Diagnostic and Interventional Radiology, University Hospital Heidelberg, Heidelberg, Germany; 25 Department of Radiology, German Cancer Research Center (DKFZ), Heidelberg, Germany; 26 Department of Diagnostic and Interventional Radiology, Medical Center – University of Freiburg, Faculty of Medicine, University of Freiburg, Freiburg, Germany; 27 Department of Obstetrics and Gynecology, University Medical Center of the Johannes Gutenberg University Mainz, Mainz, Germany; 28 Faculty of Medicine Mannheim, Department of Obstetrics and Gynecology, University of Heidelberg, Mannheim, Germany; 29 Department of Obstetrics and Gynecology, Heidelberg University Hospital, Heidelberg, Germany; 30 Department of Hematology, Medical Oncology and Pneumology, University Medical Center, Mainz, Germany; 31 University Cancer Center Mainz, Johannes Gutenberg University Mainz, Mainz, Germany; 32 Present Address: Johannes Gutenberg University, Obere Zahlbacherstr. 63, Building 911, Mainz, 55131 Germany

**Keywords:** Tumour immunology, Gynaecological cancer

## Abstract

Immunotherapy with immune checkpoint blockade (ICB) in epithelial ovarian carcinoma (EOC) shows limited clinical benefit only for a small subset of patients. Overall response rates are low, so that overcoming immunotherapy resistance and improved stratification are key. In this study, we investigated the immunometabolic landscape of EOC with a focus on omental metastases, identifying lipid-laden macrophages as central elements for actionable therapeutic vulnerabilities and giving rise to biomarkers for improved patient stratification. Using patient-derived explants, we demonstrated a functional dichotomy inside the typically lipid-rich microenvironment of omental metastases: augmented maintenance of effector T cell function, while lipid uptake and processing by tumor-associated macrophages (TAMs) induces oxidative stress–dependent signaling programs, which drive macrophage dysfunction and immune suppression. Pharmacological modulation of lipid-driven signaling pathways through CCR5 inhibition (inflammation modulation through maraviroc) or blockade of the lipid scavenger receptor CD36 reprograms TAMs, restores T cell activity, and enhances antitumor immune responses within lipid-rich tumor niches. Mechanistically, studies in humanized mouse models reveal that maraviroc-mediated CCR5 inhibition induces transcriptional programs associated with immune activation in stressed, lipid-laden human TAMs. Consistent with these mechanistic insights, we demonstrated that the specific immunometabolic niche in omental metastases is clinically associated with responsiveness to ICB. We propose a non-invasive radiomics and machine-learning–based analysis of imaging data to assess omental involvement for patient stratification.

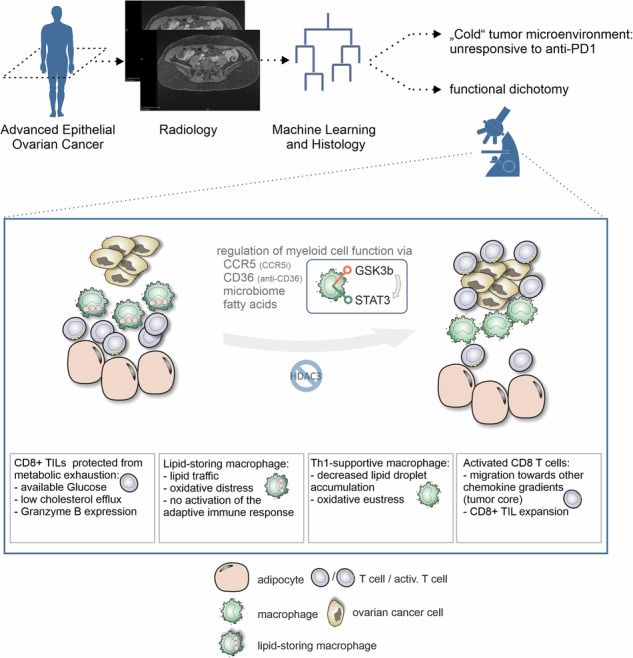

## Introduction

A large body of evidence has established an association between overall survival in patients with epithelial ovarian cancer (EOC) and the nature, composition, and localization of immune cells in the tumor.^1–4^ However, the number of current immunotherapy trials targeting EOC is limited, and the clinical results after immune checkpoint blockade (ICB) remain modest, with overall response rates between 10% and 15%.^5–7^ Potential explanations include the relatively cold immune infiltration pattern in EOC relative to other solid tumors,^8^ as well as the immunosuppressive tumor microenvironment.^9^ During the clinical course of the disease, patients exhibit a generally good sensitivity to first-line taxane-based chemotherapy and this treatment combined with surgery can potentially lead to a complete remission. Nonetheless, 70–80% of the patients relapse within 2 years. At this stage, chemotherapy is typically no longer curative, and aggressive progression of the disease limits treatment options.^10^ Notably, the rapid symptomatic progression of advanced EOC during immune checkpoint inhibition underscores the necessity for careful stratification of individuals who may benefit from this treatment.^11,12^ Identifying predictive factors for the response to immunotherapy would facilitate the selection of patients likely to benefit from immune checkpoint blockade, thereby addressing an unmet clinical need.^13^ The limited number of patients who respond to immune checkpoint blockade experience long-term benefits, underscoring the need for improved stratification.^12^

EOC spreads primarily through the peritoneal cavity, particularly the omentum, which serves as a preferred site for metastasis. This is due to various factors,^14^ including the affinity of tumor cells for milky spots and adipocytes and tumor-stroma cross-talk involving transforming growth factor-beta and interleukin (IL)-6.^15^ A thorough understanding of the role of fatty regions in tumors is crucial beyond EOC, as the close interaction between adipocytes and cancer cells has been correlated with a distinct microenvironment^16^ and poor prognosis in other cancers, such as breast cancer^17^ and colorectal cancer.^18^ For effector T cells, the concept of nutrient competition within the tumor microenvironment suggests that metabolic exhaustion of T cells is associated with limited nutrient availability. This has led to efforts aimed at revitalizing T cells by modulating their cholesterol metabolism.^19^ Limited nutrient availability in the tumor microenvironment also restricts antigen responsiveness.^20^ However, the question of whether omental metastases—rich in adipocytes—harbor a specific metabolic vulnerability that shapes the immune landscape and response to immunotherapy remains unresolved. Addressing this question is essential for understanding the immune landscape and clinical immune responsiveness in fatty tumors, such as breast and pancreatic tumors, as well as omental metastasis of EOC.

A growing body of evidence indicates that maraviroc may serve as a potential anticancer treatment. Maraviroc is a small protein inhibitor of the CC-chemokine ligand 5 (CCL5) chemokine receptor CC-chemokine receptor 5 (CCR5). In explants of colorectal cancer liver metastases, maraviroc targets tumor-associated macrophages (TAMs), leading to their in situ reprogramming into cytotoxic cells that produce reactive oxygen species and interferon-α2 (IFNα2), resulting in selective tumor cell death.^21^ In patients with metastatic, microsatellite-stable (MSS) colorectal or pancreatic cancer, maraviroc is well tolerated as a monotherapy^21^ or in combination with immune checkpoint blockade.^22^ In addition, a tumor-supporting role of the CCL5‒CCR5 axis, which is mediated by direct promotion of cancer cell proliferation and invasion, has also been observed in experimental models of pancreatic or breast cancer.^23,24^ In models of EOC, maraviroc has been shown to potentiate trabectedin cytotoxicity in vitro and control tumor growth in vivo.^25^ Together, these observations raise the possibility that omental metastases of EOC harbor a targetable macrophage-driven vulnerability that could be exploited to enhance responses to immunotherapy.

In this study, we investigated immune characteristics in omental metastases of EOC with the aim of uncovering mechanisms of resistance to immunotherapy and identifying potential strategies for patient stratification. To maximize the translational relevance of our findings, this study integrates clinical data with experimental data from a unique series of models dedicated to the study of human immune cells in a tumor context, including patient-derived explants (PDEs) and humanized mice. Clinically, we observed for the first time that omentum infiltration is a requisite for response to ICB in EOC patients. Moreover, omental infiltration can be appraised in a noninvasive fashion with radiology and machine learning. In omental metastases, more lipids are associated to better tumor-infiltrating lymphocyte abundance and fitness along with oxidative distress in TAMs. In patient-derived tumor explants, maraviroc-mediated CCR5 inhibition or Fatty acid translocase (CD36) blockade reprograms TAMs, allowing the re-allocation of tumor infiltrating lymphocytes (TILs) towards the tumor core and supporting the anti-tumor immune response in omental metastases. Biologically, TAMs exposed to lipids can show oxidative distress and activate response pathways including Histone deacetylase 3 (HDAC3)-associated stress response and ferroptosis. Maraviroc or CD36 blockade restore oxidative eustress, reestablishing their proper response to danger signals. Collectively, our findings illustrate a metabolic-inflammatory equilibrium that could be modulated to increase response rates to immunotherapy in EOC.

## Results

### EOC omental metastases display a specific immune-excluded landscape

**Fig. 1:**
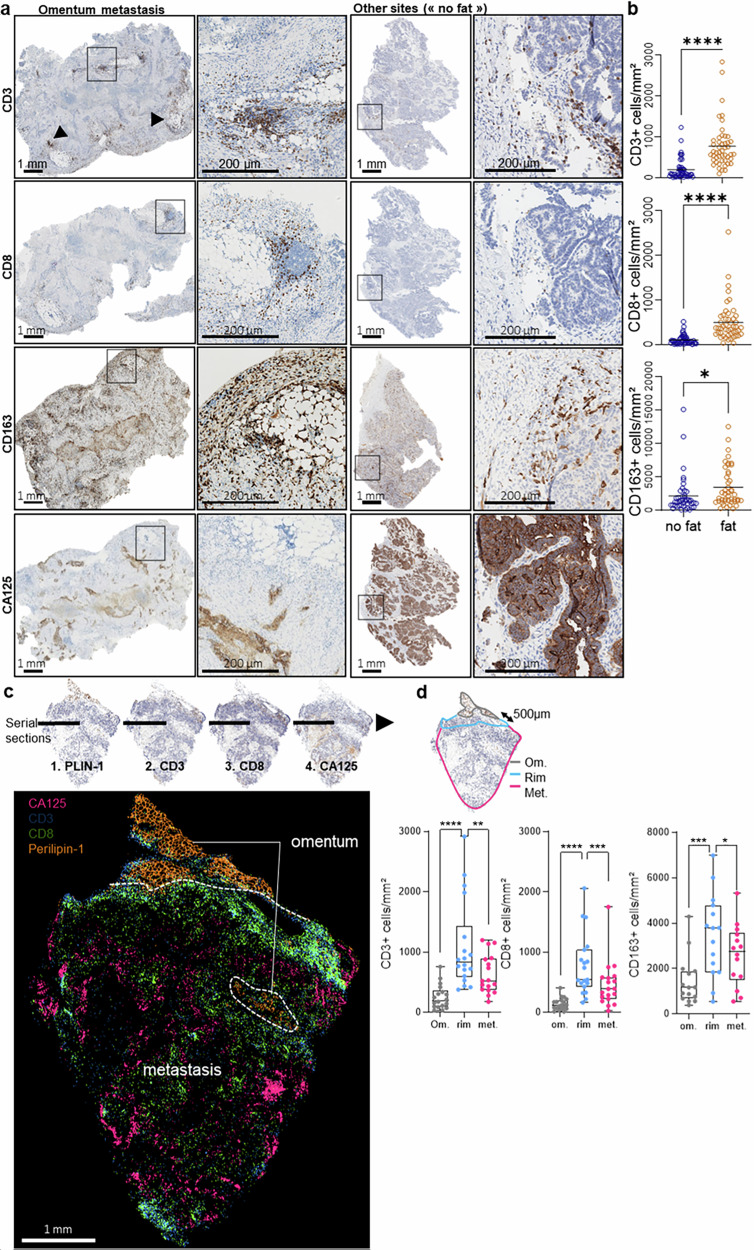
EOC omental metastases display a specific immune-excluded landscape. **a** Representative images of CD3, CD8 and CD163 staining in ovarian cancer sections. **b** respective cell density measurements based on whole-slide image analysis (*n* = 102). **c** Virtual overlay of immunohistochemistry data from four consecutive EOC sections. **d** Density of TILs in fat-containing tumors (*n* = 18), in specific parts of the tumor: fatty area (“Om.”), 500 µm-wide rim area around the fatty area (“rim”) and in the rest of the tumor (“Met”). Statistics: Two-tailed Student’s *t* test (**a**) and Wilcoxon signed-rank test (**d**)

We first studied the infiltration and spatial distribution of immune effector cells in omentum metastases of EOC. On the basis of the detectable presence of visceral adipose tissue in histological tumor sections, our cohort of 102 EOC samples (for patient characteristics, see supplementary Table 1) was separated into two groups. Tumors labeled “omentum” during surgical resection and showing visible patches of visceral adipose tissue (VAT) on histological sections (supplementary Fig. 1a) were classified as omental metastases (“fat,”, i.e., OM). These were compared to tumors labeled “primary tumor” or “peritoneal lesion” during surgery, which lacked omental tissue and showed no visible VAT histologically (“no fat”) (supplementary Fig. 1a). The presence of tumor cells was systematically confirmed by immunohistochemistry for the EOC tumor cell marker CA125, the distribution of which was similar to that of epithelial cell adhesion molecule (EpCAM) staining for all epithelial cells (supplementary Fig. 1a, b). Omental metastases were infiltrated by greater numbers of lymphocytes (CD3+ TILs), cytotoxic T cells (CD8+ TILs), and tumor-associated macrophages (CD163+ TAMs) (Fig. 1a, b) than tumor sites devoid of VAT. Omental metastases were also infiltrated by a greater density of Granzyme B + T cells and contained an elevated proportion of Tcf7+ T cells (supplementary Fig. 1c) than nonfatty tumors. However, the density of PD-1+ exhausted T cells did not differ significantly between the two groups (supplementary Fig. 1c). Notably, effector T cells in fat-containing tumors accumulate away from tumor cells and accumulate around adipose tissue areas, as illustrated by a virtual overlay of immunostaining for cancer cells (CA125), CD3 + , and CD8+ TILs (Fig. 1c). This particular distribution was confirmed by image analysis (Fig. 1d), which confirmed the significant accumulation of immune cells, including CD3+ and CD8 + T cells, along CD163+ TAMs at a 500 µm-wide tumor-VAT interface (“rim”). Together, these results indicate an abundance of T cells and their skewed distribution toward visceral adipose tissue in omentum metastases.

### EOC omental metastases display a specific inflamed landscape

**Fig. 2:**
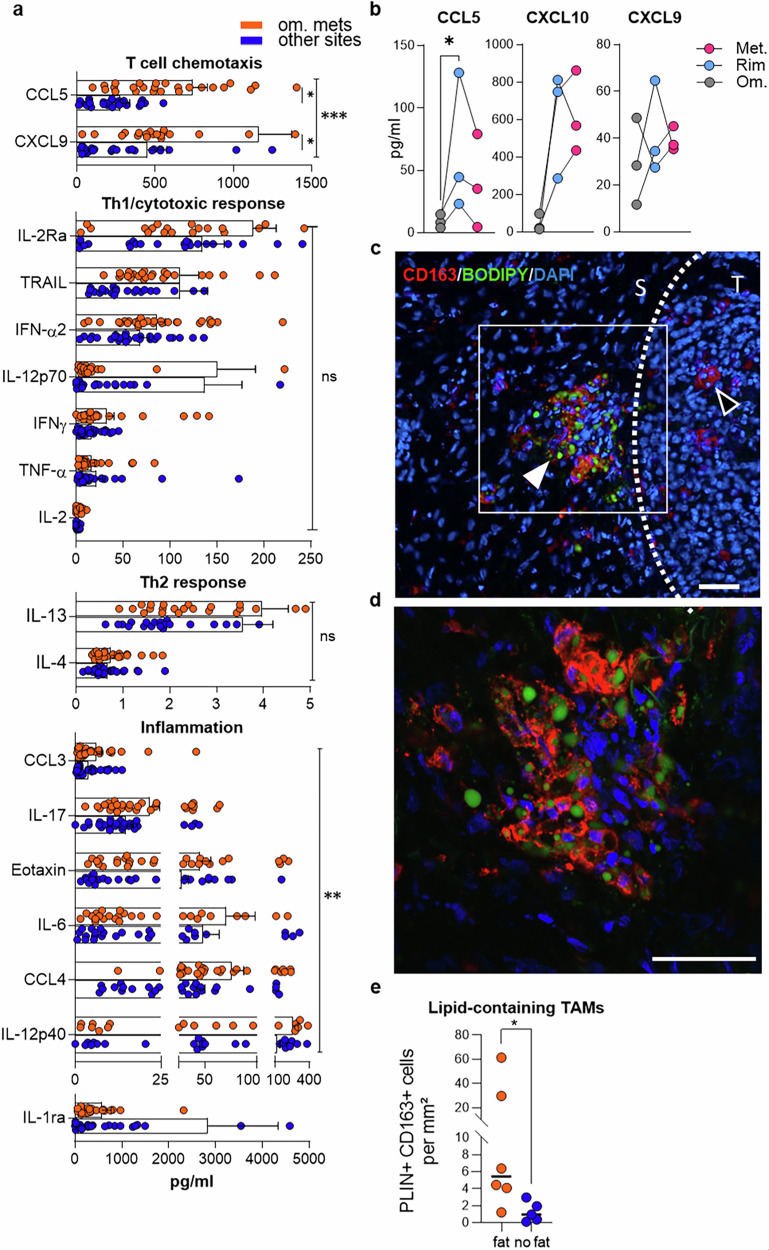
EOC omental metastases display a specific inflamed landscape. **a** Cytokine profiling of ovarian carcinomas containing VAT (*n* = 24) or not (*n* = 23). Mean ± SEM. **b** Selected cytokine concentrations in EOC samples after laser-capture microdissection into three spatial compartments, labeled “Om”, “Rim” and “Met”. **c** Microimages illustrating scavenger TAMs (CD163) containing (white arrow) or not containing (empty arrows) lipid droplets (BODIPY) in a cryosection of an omental metastasis. S: stroma, T: tumor. **d** Close-up confocal image focusing on the square drawn in panel **c**. Scale bars: 50 µm. **e** Number of perilipin+CD163+ TAMs in omentum metastases (“fat”) and in other sites devoid of visceral adipose tissue (“no fat”). Statistical tests: ANOVA (**a**), Dunn’s multiple comparison test (**b**), Mann‒Whitney test (**e**)

To further characterize the immune landscape in EOC, we conducted Luminex-based multiplex cytokine profiling on corresponding tumor lysates. In the analysis of 50 cytokines, chemokines, and growth factors, the protein concentrations of IL-2ra, TRAIL, IFN-a2, IL-12p70, IFN-g, tumor necrosis factor alpha (TNF-a), IL-2, IL-13, and IL-4 in the tumor lysates from omental metastases versus tumor sites devoid of fat were similar (Fig. 2a), despite the differences in T-cell numbers (Fig. 1a). Only two factors individually discriminated omental metastases: the T-cell chemokines CXCL9 and CCL5 (Fig. 2a). CCL5 is expressed by T cells, and accordingly, the distribution of CCL5 matches that of T cells, with a peak of CCL5 in the rim region (Fig. 2b). The other two highly concentrated T-cell chemokines, CXCL9 and CXCL10, were distributed without any obvious spatial pattern (Fig. 2b). These results were confirmed by histological analysis (supplementary Fig. 2a, b).

In addition to their T-cell chemokine signature, omental metastases specifically produce multiple inflammatory factors at relatively high concentrations while containing relatively low amounts of the anti-inflammatory factor IL-1ra (Fig. 2a). Moreover, a subpopulation of stromal scavenger CD163+ TAMs that accumulate lipid droplets (stained with the BODIPY probe for neutral lipids) was present in omental metastases (Fig. 2c, d). These lipid-containing TAMs were significantly less common at other sites when systematically analyzed by perilipin-2 (PLIN2)-CD163 immunohistochemistry (Fig. 2e).

### T cells in fat-containing tumors accumulate lipids and kill autologous tumor cells

**Fig. 3:**
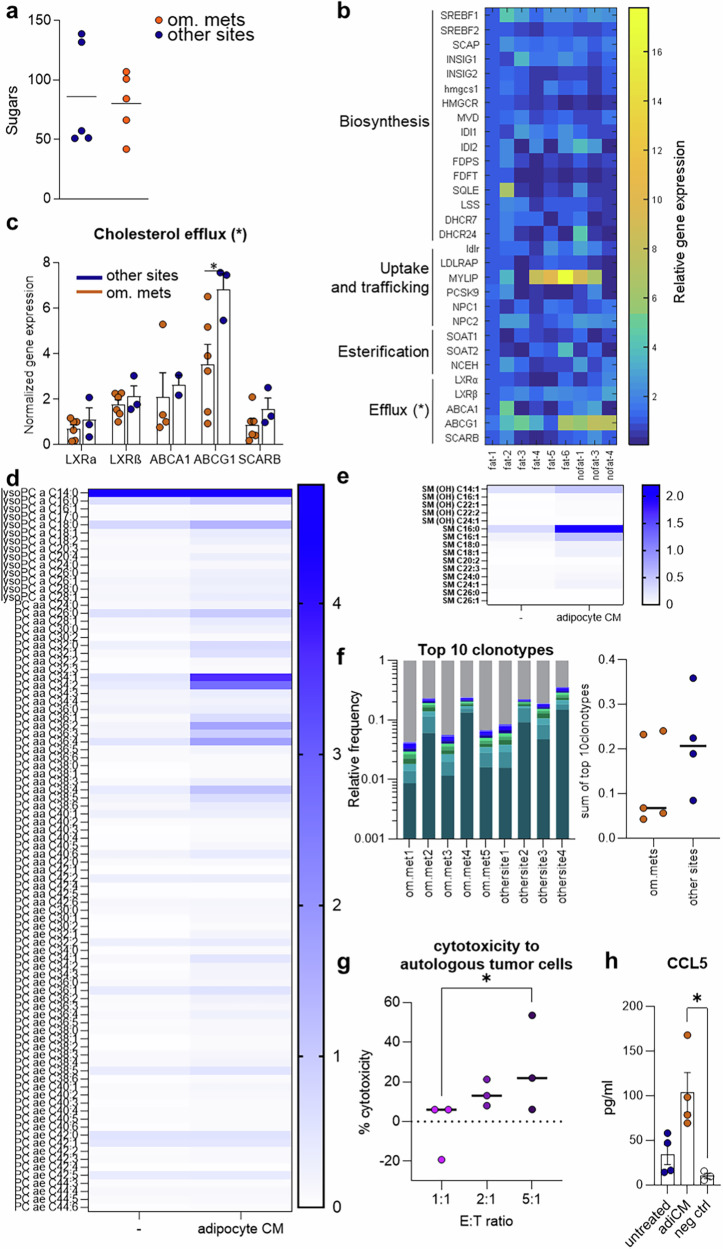
T cells in fat-containing tumors are intrinsically metabolically prone to activation. **a** Concentrations of sugars in EOC samples (measured by mass spectrometry). **b** Heatmap visualization of the relative expression of 30 genes involved in cholesterol metabolism. The Z score of the average 2^-ΔΔCT^ across CD8^+^ T cells isolated from 10 EOC samples (5 fatty tumors, 5 nonfatty tumors) is plotted. **c** Normalized expression of a subset of genes involved in cholesterol export in the abovementioned CD8^+^ TILs. Mean ± SEM. **d** Concentration of sphingolipids in TILs either untreated or treated with adipocyte supernatant. **e** Concentrations of various lipids in TILs either untreated or treated with adipocyte supernatant. **f** Percentage of the top 10 most frequent TCR CDR3 aa sequences in CD8^+^ T cells isolated from EOC samples (*n* = 10). **g** Extracellular LDH concentration as a direct measure of the cytotoxicity of TILs isolated from a fatty tumor in coculture with autologous tumor cells. E: effector cells (TILs), T: target cells (tumor cells). The results of three independent experiments are presented. **h** Extracellular concentration of CCL5 in T cells cultures in presence of conditioned medium from adipocytes (adiCM). Negative control: extracellular concentration of CCL5 in conditioned medium from adipocytes. Statistics: two-way ANOVA (**b**), unpaired t test (**c**). Kruskal‒Wallis test (**g‒h**)

Because we did not observe any elevated Th1/cytotoxic- or Th2-related cytokines in omentum metastases despite the elevated T-cell numbers (Fig. 2a), we investigated their metabolic and functional status. All tumor sites had similar glucose concentrations (Fig. 3a), potentially excluding the hypothesis that T-cell exhaustion is induced by glucose deprivation.^26^ However, CD8^+^ TILs isolated from EOCs presented differences in the expression of genes related to cholesterol metabolism: the expression of genes involved in cholesterol efflux was lower in CD8^+^ TILs from omentum metastases than in those from other sites (Fig. 3b, ANOVA, two-tailed *p* = 0.0142). Individually, ABCG1 cholesterol transporter gene expression was significantly downregulated in CD8^+^ TILs isolated from omentum metastases (Fig. 3c), indicative of metabolism consistent with T-cell activation.^27^ Finally, metabolomics profiling indicated that CD8^+^ TILs treated with adipocyte supernatants were capable of actively internalizing a variety of lipids from their extracellular environment (Fig. 3d, e). These findings suggest that T cells in omental metastases adapt to their nutrient environment by taking up locally available lipids, suggesting that lipid abundance might contribute to their increased fitness, a possibility that warrants further investigation.

To confirm that TILs isolated from omental metastases were tumor reactive, we analyzed their repertoire and capacity to kill tumor cells. TILs in omental metastases appeared to have a polyclonal expansion similar to that of TILs in fat-free tumors (Fig. 3f), and they were able to kill autologous cancer cells in vitro (Fig. 3g), confirming their effector capacity and tumor specificity.

Finally, naïve T cells cultured with CM from primary adipocytes secreted elevated amounts of CCL5 (Fig. 3h). These findings suggest that the elevated T-cell-derived CCL5 levels observed in omentum metastases (Fig. 2a, supplementary Fig. 2a) might be associated with or driven by increased lipid availability at that site.

Globally, these data indicate that abundant, tumor-reactive, metabolically fit effector T cells are present at the tumor-VAT interface.

### CCR5 blockade in fatty tumors mobilizes T cells toward the tumor core in a macrophage-dependent fashion

Because omental metastases produce large amounts of CCL5 at the tumor-VAT interface (Fig. 2a, b, supplementary Fig. 2a), we reasoned that although CCL5 is needed for T-cell chemotaxis to the tumor site, it might act as a chemokine-based trap for T cells, preventing TILs from migrating inside the tumor core. To test this theory, we blocked the CCL5‒CCR5 axis via maraviroc, a clinically approved CCR5 inhibitor. As a functional model, we used fresh ovarian cancer specimens cultured as whole-tissue explants. To this end, we tailored our previously described explant tissue culture model.^21^ to ovarian cancer tissue (supplementary Fig. 3a-c) because it faithfully maintains a stable proteome (supplementary Fig. 3d), allows for postculture spatial analysis and recapitulates the clinical response rates to immunotherapy (supplementary Fig. 3e).

**Fig. 4:**
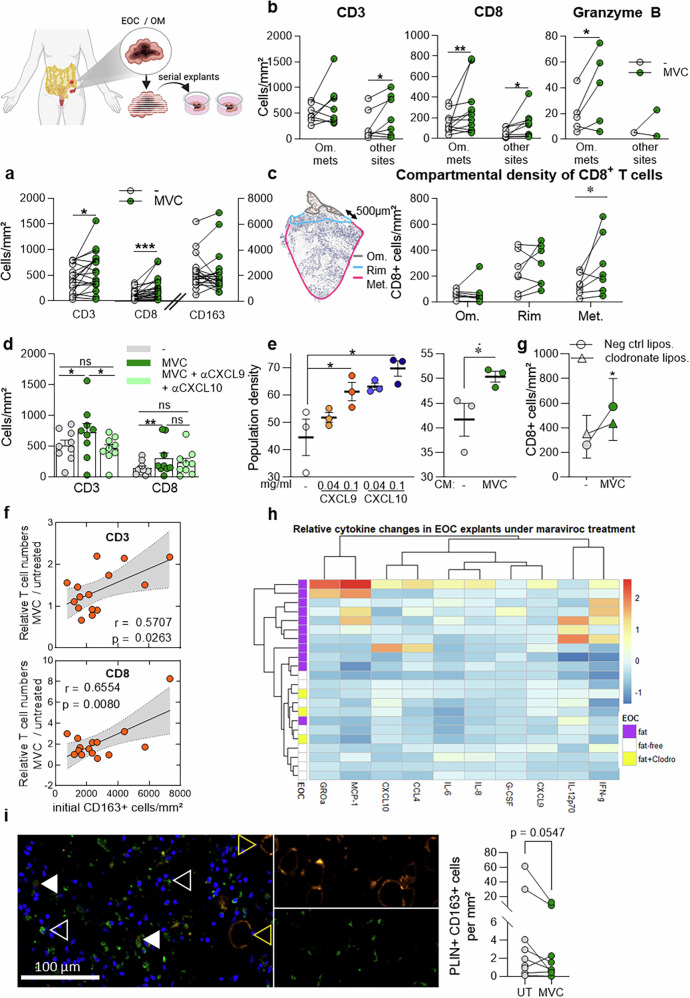
CCR5 blockade in fatty tumors mobilizes T cells toward the tumor core in a macrophage-dependent fashion. Histological density of immune cells in sections of EOC explants (cells/mm²) after 48 h of culture with maraviroc (MVC) (**a**) globally (*n* = 20) or (**b**) after separation into two groups: omentum metastases (n = 12) and fat-free tumor sites (n = 8). **c** Compartmental density of CD8-positive T cells as analyzed by immunostaining (*n* = 7). **d** Histological density of CD3+ and CD8 + T cells in EOC explants after 48 h in culture with maraviroc and with anti-CXCL9 and anti-CXCL10 blocking antibodies (*n* = 9). Mean ± SEM. **e** Proportion (%) of naïve T cells that proliferated in a CMFDA dilution assay after 7 days of treatment with either recombinant CXCL9 or CXCL10 (at concentrations of 0.04 and 0.1 µg/ml) or with CM from a fatty EOC, either untreated or treated with maraviroc. **f** Correlation between the relative changes in histological T-cell density after 48 h of treatment with maraviroc and the initial density of scavenger TAMs in EOC samples (CD163+ cells/mm²) (*n* = 15). **g** Histological density of T cells in EOC samples containing VAT after 48 h of culture with maraviroc and with an additional pretreatment with clodronate liposomes (*n* = 7). **h** Selected modifications in the cytokine concentration in the lysates of EOCs after explant culture with maraviroc. Cytokines were measured in omentum metastases (*n* = 9), fat-free tumor sites (*n* = 7) and omentum metastases after pretreatment with clodronate liposomes (*n* = 3). Statistical tests: paired *t* test. **i** Representative microimages of Perilipin-1 (PLIN1, orange)-CD163 (green) immunostaining of an explant and corresponding quantification of perilipi-positive TAMs in explants under maraviroc (statistics: Wilcoxon signed rank test)

In patient-derived explants, maraviroc-mediated CCR5 inhibition for 48 h led to an increase in the numbers of CD8+ and CD3+ TILs across the whole tissue, as assessed histologically, likely reflecting in situ proliferation (Fig. 4a). Moreover, increased Granzyme B + T-cell numbers in response to maraviroc were selectively observed in omental metastases (Fig. 4b). Spatial quantification revealed that CD8 + TIL expansion occurred specifically in the tumor core rather than at the rim or omental regions (Fig. 4c). Since no T cells were added to the explant cultures, this localized increase in CD8 + TIL numbers likely resulted from a combination of proliferation and intratumoral migration. We reasoned that maraviroc, which releases TILs from CCL5-mediated chemotactic pressure at the tumor-VAT interface, acts by allowing them to migrate toward other chemokine gradients, including CXCL9 and CXCL10, both of which are expressed abundantly in the tumor core (see Fig. 2b and supplementary Fig. 2b). Accordingly, antibody-based blockade of CXCL9 and CXCL10 reversed maraviroc-induced TIL expansion (Fig. 4d). Consistent with this expansion in tissue culture, increased proliferation of naïve T cells from healthy donors was observed upon treatment with recombinant CXCL9 and CXCL10 and upon treatment with conditioned medium from maraviroc-treated fatty EOC explants (Fig. 4e). Taken together, these results highlight a mixed response to maraviroc on the basis of both CXCL9- and CXCL10-associated proliferation and migration.

Because fibrosis is an obstacle to TIL infiltration and since CCR5 inhibition diminishes fibrosis in other tumor models,^28–30^ we also inspected whether the collagen fiber distribution is affected by maraviroc treatment in patient-derived explants. Prior to maraviroc treatment, we observed a spatial association between fibers, as observed by Masson Trichrome staining, and the omentum (supplementary Fig. 4a). There was a modest trend toward increased fibrosis in the metastases, but this increase was not significant (supplementary Fig. 4b). However, the fiber distribution in the omentum metastases was not homogeneous, with more collagen fibers present at the tumor-omentum interface (supplementary Fig. 4c–e). In patient-derived explants, maraviroc treatment significantly decreased the proportion of the tissue surface occupied by collagen fibers (supplementary Fig. 4f). The observed decrease in fibrosis could also account for the T-cell migration and expansion triggered by maraviroc treatment.

Because TAMs express CCR5 and because maraviroc has been shown to reprogram TAMs in situ in other tumors,^21,31^ we tested whether the effects on CD8+ TILs depend on macrophages. The amplitude of T-cell expansion in explants after CCR5 blockade was positively correlated with the initial number of CD163^+^ TAMs (Fig. 4f). To investigate whether the effect of maraviroc depended on TAM targeting, we pretreated explants with clodronate liposomes prior to maraviroc administration to inhibit the functions of TAMs. We first ascertained that liposomes effectively reached TAMs via the use of fluorescent liposomes (supplementary Fig. 5a). Histologically, we confirmed that the vast majority of cells positive for fluorescent liposomes were indeed CD163+ TAMs (74%), and the coverage of CD163+ TAMs was almost complete (93% of TAMs were positive for fluorescent liposomes) (supplementary Fig. 5a).

In these settings, the expansion of CD8^+^ T cells upon CCR5 blockade could not be reproduced in explants from omentum metastases, where the functional secretion of TAMs was impeded by pretreatment with clodronate liposomes (Fig. 4g), confirming that the maraviroc-mediated proliferation of TILs also relies on the number and functionality of TAMs.

Furthermore, CCR5 blockade also altered cytokine concentrations in tumors (Fig. 4h), including increased levels of proinflammatory markers (IFN-γ, TNF-α, granulocyte colony-stimulating factor, CCL2, IL-1β, IL-6, IL-8, and growth-regulated oncogene alpha). Notably, this effect was consistently observed only in omental metastases (Fig. 4h, supplementary Fig. 5b) but not in omentum metastases, in which TAM functions were inhibited by clodronate liposome pretreatment (Fig. 4h and supplementary Fig. 5c).

Finally, the proportion of lipid-containing TAMs significantly decreased in response to maraviroc (Fig. 4i). Taken together, these results suggest a functional role for maraviroc in modulating lipid contents in TAMs and potentially their inflammatory phenotype in situ, which might in turn support T-cell expansion and activation.

### CCR5 or CD36 inhibition in TAMs induces specific cytokine patterns through distinct signaling pathways

**Fig. 5:**
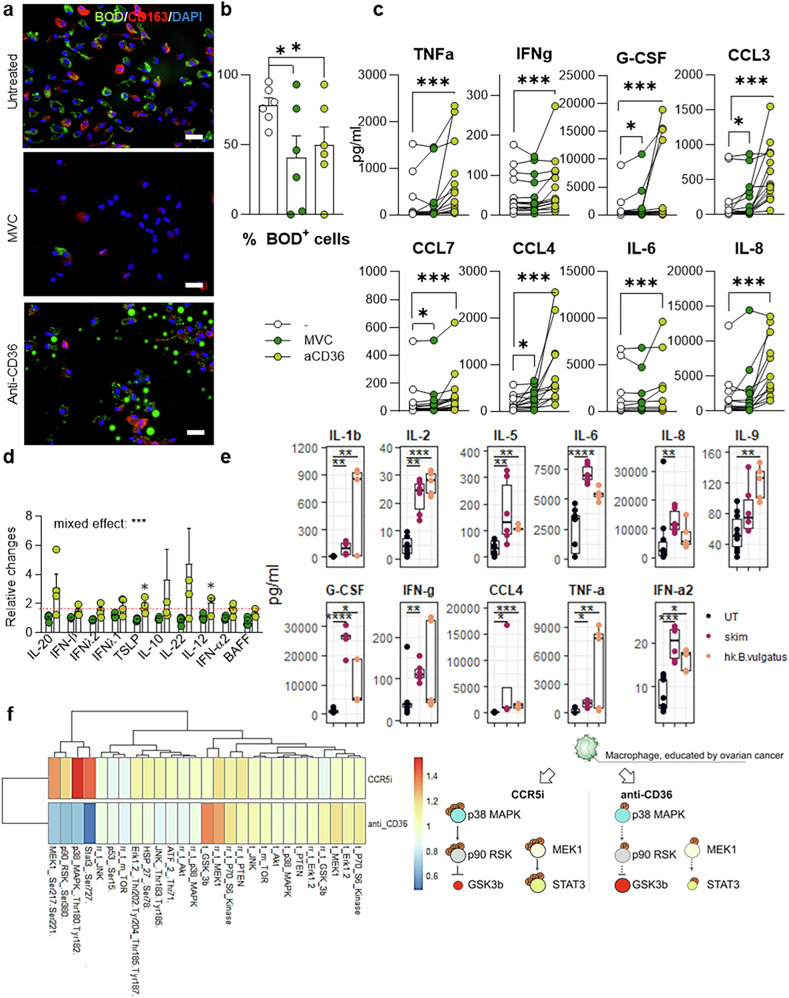
CCR5 or CD36 inhibition in TAMs induces specific cytokine patterns through distinct signaling pathways. **a** Microimages illustrating lipid droplet accumulation in TAMs isolated from malignant ascites via CD163 (red), BODIPY493/503 (green) and DAPI (blue) immunofluorescence (scale bar: 50 µm) and **b** quantification of the percentage of TAMs that contained lipid droplets after 18 h of treatment with maraviroc or anti-CD36 antibodies (*n* = 6). Mean ± SEM. Cytokine concentrations in the supernatant of TAMs after maraviroc or anti-CD36 treatment (**c** *n* = 9 and **d** *n* = 4). **e** Cytokine concentrations in the supernatant of TAMs after 18 h of fat deprivation (“skimmed”) or after treatment with heat-killed *Bacteroides vulgatus* (*n* = 4). **f** Relative phosphoprotein concentration in ovarian cancer-educated TAMs; schematic signaling effects of CCR5 vs CD36 inhibition on TAMs with modulation of STAT3, AKT, and GSK3. **f** The right panel shows the relative concentration changes as a heatmap. In the left panel, the relative concentrations of total proteins are depicted with the sizes of the rounds; red “P” circles indicate phosphorylation. Statistical tests: Dunn’s multiple comparison test

The effects of maraviroc on T-cell proliferation and activation were observed selectively in omentum metastases and were directly correlated with the initial numbers of TAMs. Moreover, T-cell expansion was abolished by clodronate-mediated TAM inhibition (Fig. 4f, g). These findings suggest that the impact of maraviroc could be linked to TAM metabolic features, such as lipid content, which appeared to decrease in situ under treatment (Fig. 4i). To model and manipulate lipid metabolism in vitro, we isolated TAMs from malignant ascites fluid. Approximately 80% of the ascites-associated macrophages (AAMs) contained lipid droplets (Fig. 5a, b), mirroring the TAM subpopulation observed in omental metastases (Fig. 2c).

Consistent with the influence of CCR5 blockade on lipid metabolism, maraviroc treatment significantly reduced the number of lipid droplets in AAMs, with near-complete depletion observed in multiple donors, suggesting the activation of lipolysis (Fig. 5a, b). To validate these findings, we inhibited CD36, a scavenger receptor responsible for long-chain fatty acid import. CD36 inhibition led to the extracellular accumulation of lipid droplets and decreased intracellular lipid accumulation, which is consistent with its known role in fatty acid import (Fig. 5a, b).

The cytokine changes induced by maraviroc in omentum-metastatic explants (Fig. 4h) were also observed in AAM cultures following both CD36 blockade and maraviroc, including increased secretion of several proinflammatory factors (Fig. 5c). In addition, CD36 blockade specifically promoted the secretion of various factors of the IFN and IL-10 families (Fig. 5d).

Similar to maraviroc and anti-CD36 treatment, depleting lipids from AAMs by skimming the ascites supernatants led to a striking increase in cytokine production (Fig. 5e), indicative of AAM activation. Notably, a comparable increase in proinflammatory cytokine secretion was also observed in AAMs exposed to a bacterial strain isolated from fresh ovarian tumor specimens (heat-killed *Bacteroides vulgatus*) (Fig. 5e).

To evaluate whether the common effects of maraviroc and anti-CD36 antibodies on AAMs arose from common early signaling pathways, we analyzed the phosphorylation events in AAMs under both treatments. CCR5 or CD36 inhibition in ovarian cancer-educated, ascites-isolated macrophages induced distinct cell signaling events, with activation of the glycogen synthase kinase 3 beta (GSK3β) pathway upon CD36 inhibition and MAPK–signal transducer and activator of transcription 3 (STAT3) signaling upon CCR5 inhibition (Fig. 5f). These data suggest that the common effects are not due to an identical, early signaling cascade.

### Fatty acid import blockade via CD36 inhibition resembles maraviroc-mediated effects on ovarian cancer explant cultures

To validate our hypothesis linking lipid content to TAM reprogramming in situ, we treated explants from EOC with a neutralizing anti-CD36 antibody. While CD36 inhibition triggered distinct signaling pathways in TAMs in vitro and modulated cytokine production by TAMs to different extents in vitro, it recapitulated the effects of maraviroc when applied to patient-derived explants. Similar to maraviroc treatment, anti-CD36 treatment promoted effector T-cell proliferation, specifically CD8^+^ TIL expansion (supplementary Fig. 5d), but this effect was again observed exclusively in omental metastases (supplementary Fig. 5e) across three spatial compartments (supplementary Fig. 5f). Additionally, anti-CD36 treatment reduced the number of perilipin+ TAMs in explants of Omentum metastases (supplementary Fig. 5g) and induced macrophage-related cytokine changes only in omental metastases (supplementary Fig. 5h, i), similar to the changes observed in AAM cultures (Fig. 5c).

### CCR5 or CD36 inhibition in AAMs alleviates lipid oxidation-related oxidative stress

As maraviroc and anti-CD36 activate distinct signaling pathways in AAMs yet induce remarkably similar effects in explants, we sought to identify the common molecular mechanisms linking lipid metabolism modification via maraviroc and anti-CD36 to TAM function. When focusing on BODIPY^bright^ AAMs, i.e., those with high levels of intracellular lipids, we observed two subpopulations: cells with a BODIPY staining pattern characterized by low maximum intensity (MI^low^) and diffuse edges (referred to as “traffic cells”) and cells with higher intensity per pixel and well-defined edges (MI^high^), termed “storage” cells (supplementary Fig. 6). We sorted these cell populations, treated them with maraviroc or an anti-CD36 antibody and analyzed them via SMART-seq2.

RNA-seq analysis confirmed that distinct subcellular lipid distributions in AAMs corresponded to differential transcriptional activities related to lipid metabolism and trafficking. The “traffic” cell subpopulation exhibited specific transcriptional activity, with elevated expression of the TAF11L gene family involved in transcription (supplementary Fig. 7a) in contrast to the storage population. The trafficking cells were enriched in several hallmark pathways, including UV response, ROS, TNFalpha signaling, the IFNγ response, IL6-STAT3 and IL2-STAT5 signaling, apoptosis, peroxisome and adipogenesis, in contrast to the storage population (supplementary Fig. 7b, c, Traffic versus Storage, “TvsS”). The trafficking cells also had an increased response to oxidized phospholipids (supplementary Fig. 7d) and increased general HDAC activity (supplementary Fig. 7c). In trafficking cells, HDAC3 activity was most significantly modulated (supplementary Fig. 7e), but HDAC3 expression was not (supplementary Fig. 7f). Thioredoxin reductase 1 (TXNRD1), which can be indirectly modulated by HDAC3 in cases of oxidative stress, was significantly elevated (supplementary Fig. 7f) and was the most upregulated gene in trafficking cells. HMOX1 (heme oxidase 1), which is involved in heme metabolism in response to oxidative stress, was also elevated, although not significantly. Finally, trafficking cells were enriched for GPNMB (glycoprotein nonmetastatic melanoma protein B), a protein enriched in the case of lysosomal stress, which has been associated with the protumor functions of macrophages in glioblastoma.^32^

**Fig. 6:**
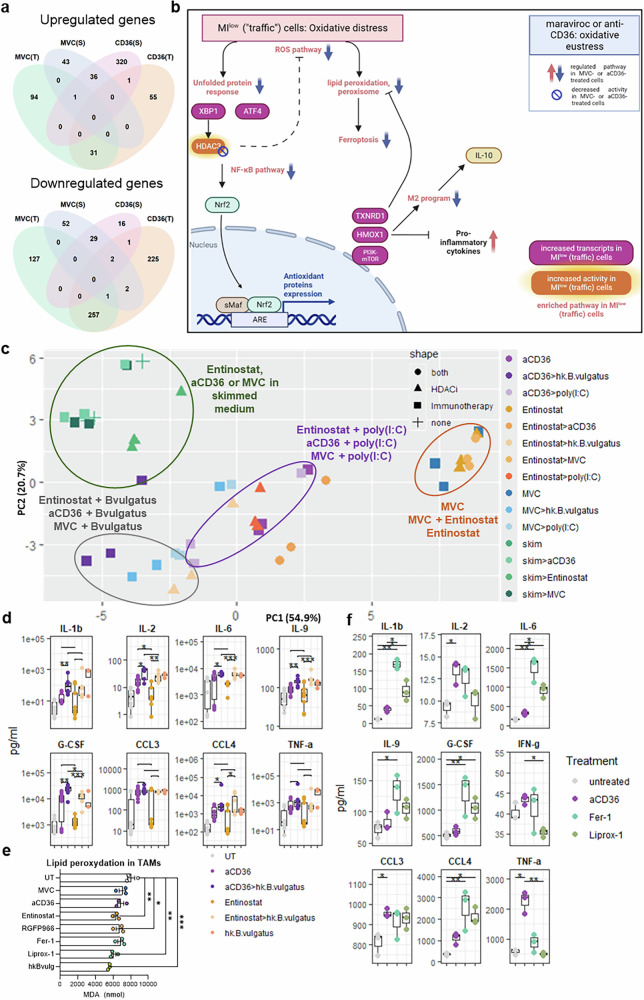
CCR5 or CD36 inhibition in AAMs relieves lipid oxidation-related oxidative distress in cells**. a** Venn diagrams illustrating the overlap in transcripts significantly modulated by maraviroc treatment or anti-CD36 treatment in trafficking cells (T) or storage cells (S) (versus the parental population). **b** Visual scheme summarizing the various transcripts, pathways and molecular activities in trafficking cells or in cells treated with either maraviroc or anti-CD36. All pathways and transcripts enriched in trafficking cells are shown in orange and purple, respectively. All pathways and transcripts repressed under both maraviroc and anti-CD36 treatments in all cells are marked with a downward arrow. In addition to HDAC3, both maraviroc and anti-CD36 treatments repress its targets. **c** Principal component analysis of the concentrations of 47 soluble factors in the supernatants of the AAMs after various treatments. The triplicate results of one representative experiment out of two are shown. **d** Concentrations of selected soluble factors used for the PCA shown in **c** in the supernatants of the AAMs (*n* = 4). Statistical test: paired *t* test. **e** Evaluation of lipid peroxidation in TAMs via measurement of the malondialdehyde (MDA) concentration in TAM lysates. Statistics: Dunnett’s multiple comparisons test. **f** Concentrations of selected soluble factors secreted by TAMs (*n* = 3, mean ± SEM). Statistics: Dunn’s multiple comparison test

When all the cells were analyzed globally, maraviroc and anti-CD36 treatments led to specific gene modulation (Supplementary Fig. 8a, b) with common gene set suppression (Supplementary Fig. 8c). Interestingly, however, both maraviroc and anti-CD36 treatment equally repressed hallmark pathways enriched in trafficking cells, including UV response, reactive oxygen species, oxidative phosphorylation, heme metabolism, adipogenesis, and PI3K‒mTOR pathways, as well as the fatty acid metabolism pathway (supplementary Fig. 8d). Both treatments also equally repressed the response to oxidized phospholipids (supplementary Fig. 7c). These results suggest that maraviroc and anti-CD36 both repress the trafficking state of macrophages. The response to either treatment appeared to be determined more by the initial cell state than by the treatment itself (Fig. 6a, Supplementary Fig. 8e). Maraviroc and anti-CD36 treatments also affected HDAC activity (Supplementary Fig. 7d, e).

Based on these results, we generated a model of TAMs treated with maraviroc and anti-CD36 (Fig. 6b). Traffic cells suffer from oxidative distress, which is characterized by enrichment of ROS, the UPR, and NF-kB, which leads to, or is associated with, the activation of an M2 program, heme metabolism as a (futile) stress response, ultimately leading to lipid peroxidation and ferroptosis. This response is associated with HDAC3 activity. Maraviroc and anti-CD36 decreased the cellular lipid content and fatty acid metabolism, which decreases oxidative stress, as evidenced by the suppression of all the aforementioned pathways. This is associated with HDAC3 target repression and M2 program repression, allowing efficient secretion of proinflammatory cytokines.

Given that HDAC3 activity is repressed significantly under maraviroc or andi-CD36 treatment, we tested whether HDAC3 activity was a bystander or a functional hub linking lipid processing and TAM dysfunction. For this purpose, we stimulated AAMs with various activation signals, including a TLR agonist (poly(I:C)), heat-killed *Bacteroides vulgatus*, or lipid-depleted culture medium (“skim”), and we treated them with maraviroc, anti-CD36, or entinostat, a selective class I HDAC inhibitor. Then, the concentrations of 50 cytokines, chemokines and growth factors in the supernatant were analyzed. Principal component analysis revealed that cells treated with entinostat clustered closely with the corresponding maraviroc- or anti-CD36-treated samples, suggesting that the effects of class I HDAC inhibition mimic maraviroc- or anti-CD36-mediated lipid depletion (Fig. 6c). Figure 6d shows a few examples of soluble factors modulated similarly by these treatments. Because entinostat targets all class I HDACs, we also treated cells with a selective HDAC3 inhibitor (RGFP966), and similar results were observed (supplementary Fig. 9a, b). Notably, while entinostat-treated cells clustered most closely with anti-CD36-treated cells (Fig. 6c), RGFP966-treated cells clustered most closely with maraviroc-treated cells (supplementary Fig. 9a), as illustrated by individual factors (supplementary Fig. 9b). This pattern aligns with the SMART-seq data: maraviroc-treated cells strongly repressed HDAC3 targets (NES = −1.421, *p* = 0.013) but did not significantly repress HDAC1 (NES = 1.328, *p* = 0.06), whereas anti-CD36-treated cells repressed HDAC3 and HDAC1 target genes in a similar manner (NES = −1.421 and *p* = 0.027, respectively; NES = −1.661 and *p* = 0.002, respectively).

To substantiate these results, we measured lipid peroxidation in AAMs in vitro. Maraviroc and anti-CD36 treatments slightly decreased lipid peroxidation in the AAMs. Entinostat and RGFP966 similarly decreased lipid peroxidation to a similar extent as exposure to activation signals (heat-killed *Bacteroides vulgatus*) or ferroptosis inhibitors (Fig. 6e). Finally, the changes in cytokine secretion in anti-CD36-treated cells were similar to those induced by ferrostatin-1, a strong ferroptosis inhibitor (Fig. 6f).

Together, these results validate the pivotal role of lipid metabolism and oxidative distress in shaping TAM responses to surrounding danger signals, underscoring the therapeutic potential of targeting lipid processing pathways to modulate immune function in the TME.

### Maraviroc reprograms stressed human TAMs in vivo

**Fig. 7:**
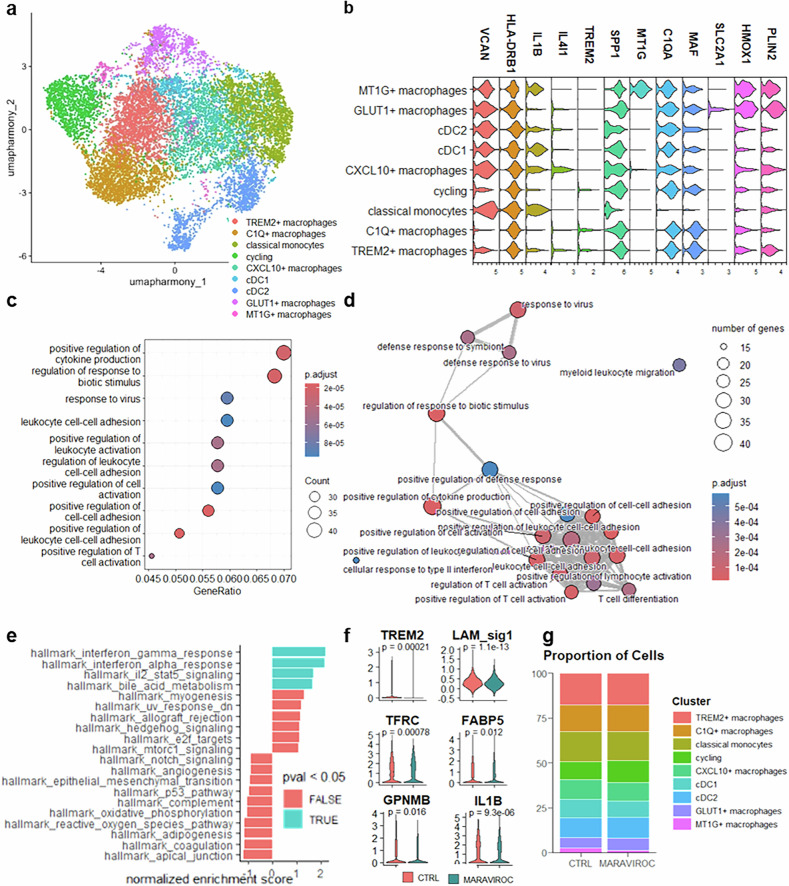
CCR5 inhibition reprograms human TAMs in vivo in a humanized mouse model. ScRNA-sequencing analysis of human myeloid cells infiltrating MDA-MB-231 tumors in MISTRG mice. **a** Clustering and annotation of myeloid cells. **b** Gene expression of TAM-specific markers in the different clusters. Enrichment analysis of the DEGs in Maraviroc-treated mice (GO enrichment analysis of biological processes) displayed as **c** enrichment plots and **d** enrichment maps. **e** Hallmark pathways identified via gene set enrichment analysis (GSEA) of genes modulated by maraviroc treatment. **f** Expression of individual genes under untreated and Maraviroc-treated conditions. **g** Proportion of myeloid cells per harmony cluster. Statistics: *t* test

We next sought to assess the impact of maraviroc on the tumor immune microenvironment in vivo via a humanized mouse platform. These mice (MISTRG strain^33^) express human genes encoding growth factors (M-CSF, GM-CSF, and IL-3) that support efficient development of the human myeloid lineage. MISTRG mice were engrafted with fetal CD34+ hematopoietic stem cells to recapitulate the human myeloid compartment.^33^ Orthotopic tumors were established by injecting MDA-MB-231 cells into the mammary fat pad. We then performed scRNA-seq on human CD45+ tumor-infiltrating immune cells, confirming the presence of both lymphoid and myeloid populations (supplementary Fig. 10). Within the myeloid compartment, we identified and annotated distinct clusters: conventional dendritic cells (cDCs), VCAN + CX3CR1 + HLA + IL1B+ classical (inflammatory) monocytes, cycling cells, and different states of macrophages. These included CXCL10+ (IL4I1+) TAMs with an interferon signature, triggering receptor expressed on myeloid cells 2+ (TREM2+) osteopontin (SPP1+) TAMs enriched for phagocytosis and lipid metabolism genes, MT1G+ TAMs with a tissue maintenance signature, C1Q + musculoaponeurotic fibrosarcoma oncogene homolog (MAF+) TAMs, and glucose transporter 1 (GLUT1)+ TAMs displaying hypoxia and a cellular stress signature, including HMOX1 (Fig. 7a, b). The projections of these clusters on the Monocyte and Macrophage Valied Resource of Single-cell Expression dataset strongly overlapped with those on the human reference dataset, indicating that this model faithfully recapitulates the spectrum of monocyte-to-macrophage differentiation states.

Maraviroc treatment induced functional reprogramming of tumor-infiltrating myeloid cells at the global level, including positive regulation of cytokine production, the adaptive immune response and antiviral response programs (Fig. 7c, d). In addition to the enrichment of hallmark interferon response pathways and the IL-2-STAT5 signaling axis (Fig. 7e), we also observed a nonsignificant trend toward the suppression of reactive oxygen species and UV-response gene sets (Fig. 7e), suggesting reduced oxidative stress, as also observed in patient-derived TAMs. Furthermore, tumor-infiltrating myeloid cells in maraviroc-treated mice presented decreased lipid signatures and TREM2 and GPNMB expression and lower levels of transferrin receptor (TFRC) and fatty acid-binding protein 5 (FABP5), which are involved in ferroptosis and lipid metabolism, respectively. Conversely, IL1B expression was increased (Fig. 7f), corroborating the observations in patient-derived TAMs. The proportions of different myeloid cell populations were rather unaffected by maraviroc treatment (Fig. 7g).

**Fig. 8:**
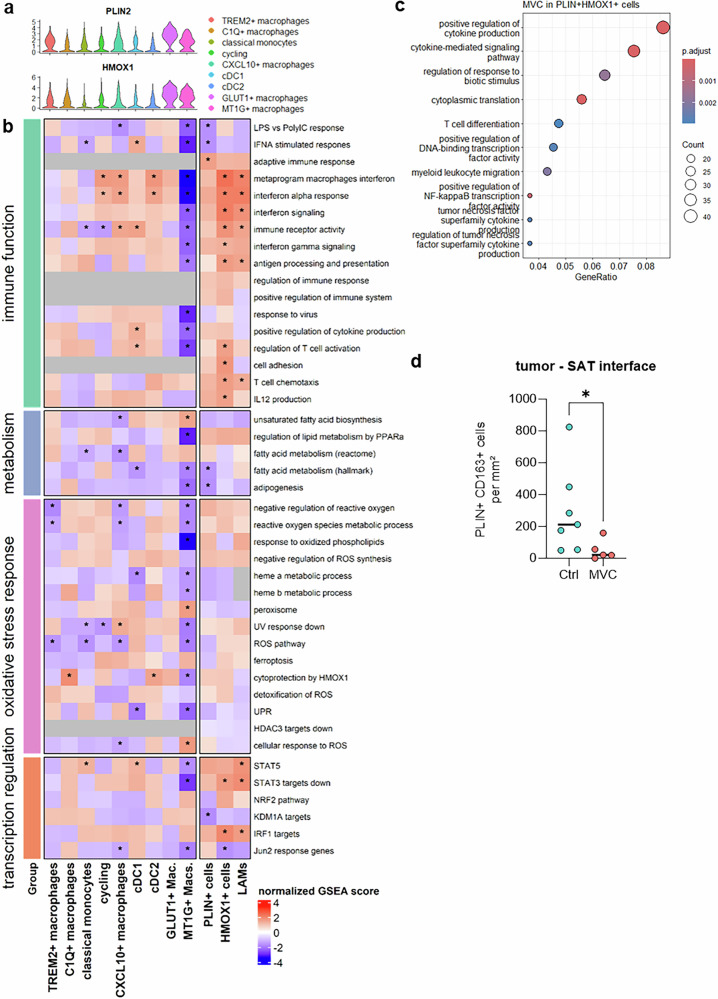
Lipid content and cellular stress affect the response of human TAMs to CCR5 inhibition in vivo. **a** Perilipin-2 (PLIN2) and HMOX1 individual gene expression across clusters in myeloid cells infiltrating MDA-MB-231 tumors. **b** GSEA scores of various pathways affected by maraviroc treatment in corresponding cell populations and in PLIN+ cells, HMOX1+ cells or lipid-associated macrophages (LAMs), i.e., cells positive for the LAM signature as defined by Kloosterman et al.^32^ **c** GO enrichment analysis of gene expression in HMOX1 + PLIN2+ tumor-infiltrating cells in mice treated with maraviroc. **d** Number of PLIN2 + CD163+ tumor-infiltrating myeloid cells at the 500 µm interface between the xenograft and adjacent subcutaneous adipose tissue (SAT)

To determine which monocyte or macrophage population drove immune activation under maraviroc treatment, we performed GSEA on each cluster separately. PLIN2 and HMOX1 were expressed across several clusters; both were particularly elevated in GLUT1+ and MT1G+ macrophages but also in CXCL10+ macrophages and TREM2+ macrophages and remained detectable in all other subsets (Fig. 8a). When each cluster was analyzed individually, none fully reproduced the maraviroc-induced effects observed in the global myeloid population or in patient-derived TAMs. However, HMOX1+ macrophages across clusters presented significantly increased immune functions in response to maraviroc (Fig. 8b). These cells also displayed trends toward reduced lipid metabolism signatures and attenuated oxidative stress response pathways, including downregulation of the UPR and of HDAC3 targets. The LAM signature (as defined by Dan Kloosterman et al.^32^) is also a determinant of response and is correlated with increased immune activity, whereas PLIN2 expression alone is not (Fig. 8b). The transcription regulators associated with these effects include increased STAT5 activity together with the repression of STAT3 and Jun2. Focusing specifically on HMOX1 + PLIN2+ tumor-infiltrating myeloid cells, we observed functional enrichment in functions consistent with maraviroc-mediated TAM activation, including TNFa production, cytokine secretion and NF-kB pathway activation (Fig. 8c). Finally, at the interface between the tumor and the subcutaneous adipose tissue, we observed a number of PLIN2 + CD163+ TAMs, which were much less numerous under maraviroc treatment (Fig. 8d), reminiscent of the observations in patient-derived explants.

These results validate the role of CCR5 inhibition in reprogramming stressed, lipid-containing TAMs in vivo.

### Proximity of tumor cells to compartmental adipose tissue as a predictor of immune checkpoint blockade response in EOC patients using noninvasive methods

**Fig. 9:**
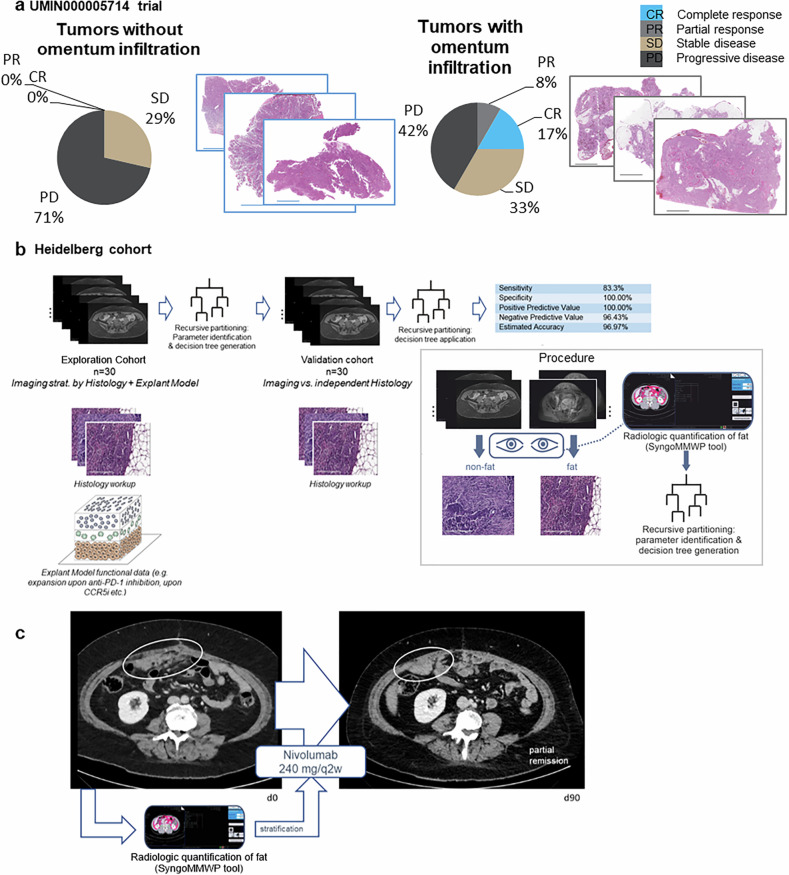
Proximity between tumor cells and compartmental adipose tissue can be predicted with noninvasive methods and modulates the response to immune checkpoint blockade in patients with EOC. **a** Graphical representation of the analysis of the clinical outcome in the UMIN000005714 clinical trial after separation into two groups according to whether VAT was present in the H&E slides before treatment (*n* = 20 patients in total). **b** Segmentation of MR images to predict the close proximity between the tumor and VAT in EOC patients and histological confirmation. The box illustrates the procedure that was followed. Radiological quantification of total and visceral body fat content is used to predict the close proximity between tumor cells and VAT. **c** Medical images illustrating partial remission in a patient after response prediction via the algorithm illustrated in a and treatment with nivolumab

Next, we sought to validate our findings clinically. There is no clinical trial of maraviroc in EOC. Therefore, we performed a retrospective analysis of the phase II UMIN000005714 trial in which carboplatin-resistant EOC patients received nivolumab after surgical resection.^5^ Blind histological assessment of H&E slides from the tumors revealed that all patients who responded to nivolumab had omental metastases prior to treatment, confirming that stratification of patients according to the close proximity of tumor cells and VAT has clinical predictive value (Fig. 9a). The group of patients in which VAT was visible in tumor sections before treatment presented a disease control rate of 25%, with 8% partial response and 17% complete response (Fig. 9a).

The next question was whether such proximity at the histological level could be predicted via noninvasive methods. With a training cohort of *n* = 30 patients, we used imaging data, histology (presence of VAT as assessed in the pathology report), and tumor explant results (indicative of whether immunotherapy induced an expansion in T cells) to generate a model predictive of the histology associated with such a response to immunotherapy. On the basis of radiology data, a validation cohort of 30 patients, comprising imaging data and pathology reports, verified this prediction (Fig. 9b). On the basis of the quantified areas and volumes of total body fat and visceral body fat, we were able to generate a decision tree predictive of the response to immunotherapy (Supplementary Fig. 11). This decision tree was applied to the decision regarding therapy for a patient with ovarian cancer. The patient, aged 53 years, had microsatellite stable (MSS) serous ovarian carcinoma and had already received all standard-of-care treatments. On the basis of the analysis of the CT scan images, the decision tree indicated a likely response to immunotherapy. Consequently, the patient received nivolumab and achieved partial remission (Fig. 9c).

These results indicate that the presence of omentum metastases might serve as a biomarker predictive of the response to immunotherapy in EOC.

## Discussion

The significant variation in the survival rates of EOC patients on the basis of the abundance, localization, and activation of T cells^2^ has prompted numerous (pre)clinical studies on immunotherapy, with little success to date.^34^ Our study focused on the omentum, a preferred site for intraperitoneal dissemination of EOC. Previous studies have emphasized the contribution of direct crosstalk between cancer cells and adipocytes to malignant progression^35,36^ and chemotherapy resistance,^37^ along with the acquisition of aggressive traits by cancer cells in melanoma,^38^ breast cancer,^39^ and ovarian cancer.^40^ Similarly, the intake of select fatty acids increases the risk of EOC.^41^ Here, we show that the specific fatty environment of omentum metastases also affects the immune landscape of tumors, creating a distinct microenvironment.

We observed TIL abundance in omentum metastases, which was consistent with the findings of a previous report in 22 EOC patients.^42^ However, poor infiltration of TILs into the tumor core and their accumulation at the omentum interface (consistent with our previous findings^8^) pose a challenge, as intraepithelial TILs are critical for predicting clinical outcomes.^2^

The TILs in omental metastases appear to be active. This is evidenced by the downregulation of genes associated with cholesterol efflux,^19,43^ their production of granzyme B, their ability to kill autologous cancer cells ex vivo, and their capacity to take up extracellular lipids, which are known to sustain T-cell metabolic fitness and contribute to the success of cellular immunotherapy in experimental models of EOC.^44^ Finally, they are enriched in Tcf7+ progenitor cells which are also associated with immunotherapy outcomes in EOC models.^45^

Metastatic cancer cells often increase glycolysis and glutaminolysis to support de novo fatty acid synthesis for biomass production, which can deplete glucose and limit T-cell function. However, in lipid-rich microenvironments such as the omentum, cancer cells adapt by importing extracellular fatty acids rather than relying on glucose-derived lipids.^36,46^ This shift in nutrient utilization likely explains why we observed glucose available at levels comparable to those in primary tumors. However, a spatially resolved analysis would be needed to exclude the possibility of T-cell exhaustion due to glucose deprivation.^26^

Overall, TILs in omentum metastases present a series of hallmarks of immunotherapy responsiveness, including their abundance and functional state, but not their spatial pattern and distribution.^47^ We leveraged this latter characteristic by neutralizing the CCL5‒CCR5 axis, a key factor for cytotoxic T-cell migration to tumors.^48^ Consistent with other reports,^23,24^ we detected elevated CCL5 levels in omental metastases, which coincided with the CD8 + T cells that produce CCL5. CCR5 inhibition with maraviroc treatment alleviated CCL5-mediated chemotactic pressure at the tumor-omentum interface, which resulted in T-cell expansion and migration to the tumor core through a CXCL9- and CXCL10-dependent mechanism.

Immune cells of the myeloid compartment may become dysfunctional when exposed to oxidized lipids. Specifically, the cross-presentation capability of dendritic cells is compromised by oxidized lipids,^49^ and studies involving XBP1 activation and deletion in mice indicate that dendritic cells experiencing ER stress do not effectively support antitumor T cells.^50^

Our observations connect lipid abundance in omental metastases to lipid accumulation in scavenger macrophages and the deregulation of lipid metabolism. A reduction in the lipid content in TAMs by maraviroc or anti-CD36 treatment reprograms TAMs, contributing to TIL proliferation and activation. Similarly, either depletion of fatty acids in the medium or activation of TAMs with heat-killed bacteria led to comparable changes in cytokine production. The connection between TAM properties and lipid transport aligns with recent reports linking fatty acid metabolism and M2 function in other models.^51,52^ The metabolism of long-chain fatty acids is critical for macrophage M2 polarization^53^ and for the immunosuppressive and protumorigenic functions of TAMs.^54–56^

In lipid-trafficking AAMs, we observed transcriptional pathways associated with oxidative stress, which are known to contribute to their tumor-supportive functions.^55^ Lipid-trafficking AAMs express elevated amounts of AT4, a transcription factor involved in the stress-induced unfolded protein response,^57^ similar to findings in dendritic cell EOC mouse models^50^ and in TAMs in melanoma-bearing mice.^56^ Oxidative stress in macrophages can also induce the activity of HDAC3, which triggers a transcriptional and nontranscriptional program that mitigates oxidative stress. HDAC3 and XBP1 activate the transcription of heme-oxidase-1 (HMOX1) through the Akt-mTORC2-Nrf2 pathway.^58^ HMOX-1 contributes to ROS control through haem degradation.^59^ We observed elevated HDAC3 activity in lipid-trafficking AAMs, along with enrichment of the Akt-mTOR pathway and elevated levels of HMOX1 and ATF4 transcripts. Furthermore, lipid-trafficking AAMs overexpress thioredoxin reductase 1 (TXNRD1) and activate lipid peroxidation and ferroptosis, indicating uncontrolled ROS distress.^60^

We substantiated these results in the humanized MISTRG mouse model, which enables the development of a human myeloid compartment and faithfully recapitulates the spectrum of macrophage differentiation states.^33^ Maraviroc induces transcriptional reprogramming of tumor-infiltrating myeloid cells, including reduced expression of protumor markers and genes involved in lipid metabolism and ferroptosis, together with increased cytokine production. These changes are associated with attenuation of oxidative stress programs, downregulation of the UPR, and increased interferon and IL-2–STAT5 signaling. Notably, HMOX1+ cells across multiple subsets of myeloid cells (including macrophages and dendritic cells) display the strongest immune activation signature, indicating that this population may be a critical determinant of response.

The convergence of these effects with those observed in patient-derived AAMs underscores the translational relevance of CCR5 blockade as a strategy to rewire macrophage states in the tumor microenvironment.

Thus far, our results cannot formally exclude a contributing role of dendritic cells in the maraviroc- or anti-CD36-mediated effects on TIL activation observed in situ. Dendritic cell clusters (cDC1s and cDC2s) also express CCR5 and CD36 and respond to maraviroc in vivo. In patient-derived explants, a minor proportion of the increased cytokine production might also originate from activated DCs. Additionally, the targeting of TAMs by clodronate liposomes, although very sensitive, is not entirely specific, as CD163-negative cells, most likely DCs, also phagocytose liposomes. Nevertheless, the in vitro experiments on pure TAM populations, the overwhelming abundance of TAMs in human specimens,^61^ along with the positive correlation observed between TAM numbers in tissue and TIL expansion under maraviroc, jointly make this hypothesis unlikely.

Our findings highlighting the presence of omental metastases at treatment onset to predict the response to immunotherapy in EOC patients are supported by a retrospective analysis of the UMIN00000574 trial. The ability to determine the occurrence of omental metastases through a noninvasive method further demonstrates the practicality of radiology-guided patient stratification, as emphasized by one prospective case, although a more robust clinical validation with multiple prospective cases is needed for further development. Finally, while omentum involvement may be a necessary condition for a response in this context, it is not sufficient on its own to guarantee a response to immune checkpoint blockade. Additional immunological or molecular factors, including but not limited to the CCL5‒CCR5 axis, likely modulate the responsiveness to checkpoint blockade in this patient population.

Overall, our findings revealed that the proximity of visceral adipose tissue has opposing effects on immune cells. TILs appear protected from metabolic exhaustion and demonstrate increased sensitivity to immune checkpoint blockade, whereas TAMs are dysfunctional owing to increased lipid trafficking and consequent oxidative stress. Nonetheless, their functionality can be restored in situ, enabling the transition from an ‘immune-excluded’ to a ‘hot’ tumor. This study highlights the relationship between lipid metabolism and (antitumor) inflammation in the innate and adaptive immune systems, enhancing our understanding of the interactions among the cellular components that influence the microenvironments of adipose and adjacent tissues. A limitation of our study lies in the size of the retrospective analysis and the unique prospective design. Nonetheless, our work offers proof of feasibility and a rationale for patient stratification for potential benefits from immunomodulatory therapies, including CCR5 inhibition or other macrophage-modulating treatments.

## Materials and methods

### Study design

The aim of this study was to identify the immune landscape of omental metastases of EOC and identify actionable factors for immunotherapy. Our study includes a characterization of the immune landscape of omentum-infiltrating tumors (described as “fatty”) compared with that of primary tumors or non-omentum-infiltrating metastases, functional intervention in patient-derived explants of EOC, functional intervention in tumor-associated macrophages isolated from malignant ascites and RNA sequencing.

For PDEs, the sample size was determined via the R package pwr (version 1.3—0). The *p* value was set at 0.05, and the power was set to 0.75. The effect size was determined via prior PDE experiments.

### Patient material and tissue explant culture

All experiments were conducted in accordance with the Declaration of Helsinki, the International Ethical Guidelines for Biomedical Research Involving Human Subjects (CIOMS), the Belmont Report, and the U.S. Common Rules. Tumor samples were obtained from Women’s Hospital (Heidelberg University Hospital) with the approval of the ethical board of the University of Heidelberg (S-22/2013 and S-507/2019). Material of the biobank was collected with approval of the ethics committee of the University Medical Center Mainz. Written consent was obtained from all patients.

After surgical resection, the tumors were rapidly processed for culture, transported in saline solution on ice, and chopped into small pieces (approximately 1 × 5 × 5 mm). One fragment was directly cryopreserved in the OCT embedding matrix, and the other fragment was directly fixed in formalin and embedded in paraffin. The remaining explants were cultured in a 24-well plate with constant gentle agitation via Whitley H35 fluorescence (Whitley, Shipley, UK) at 37 °C with 15% O_2_ and 5% CO_2_. The culture medium was MEM (Thermo Fisher Scientific) containing 1% L-GlutaMAX (Thermo Fisher Scientific). Treatment was added immediately at culture onset (except for treatment combined with liposomes, which were added 2 h later), and the cultures were incubated for 48 h if not otherwise stated. After culture, the tissue explants were cryopreserved, formalin fixed, and paraffin embedded (Supplementary Fig. 3a). All explants were subjected to careful quality control before further experimentation. Quality controls (supplementary Fig. 3b–d) included visual inspection of tissue integrity (as H&E; hematoxylin‒eosin), confirmation of the tumor cell content (a minimum of 20% of the whole section area positive for the tumor marker CA125, see histology section), and confirmation of molecular stability (see multiplex cytokine analysis).

### Immunohistochemistry, virtual microscopy and image analysis

Three-micron-thick consecutive sections were cut from the paraffin-embedded tissues. Immunostaining was performed via a Bond-Max automated stainer (Leica Biosystems, Nussloch, Germany) and a DAB-based Polymer Refine Detection Kit (Leica Biosystems). The primary antibodies used were anti-CA125 (clone Ov185:1, Novocastra; RRID: AB_442044), anti-CD3 (clone Sp7, Abcam; RRID: AB_443425), anti-CD8 (clone 4B11, Novocastra; RRID: AB_442068), anti-CD163 (clone EDHu-1, AbD Serotec; RRID: AB_2074539), anti-granzyme B (clone 23H8L20, Life Technologies; RRID: AB_2532478), anti-PD-1 (clone NAT105, Abcam; RRID: AB_881954), and anti-FoxP3 (clone 236 A/E7; eBiosciences; RRID: AB_467555). Antigen retrieval was performed via Epitope Retrieval solutions 1 and 2 (Leica Biosystems) or Fast enzyme (Zytomed, Berlin, Germany) if necessary. Masson’s trichrome staining was performed via a trichrome staining kit (Sigma‒Aldrich). For fluorescent immunostaining, the following antibodies were used: CD163 (clone EDHu-1, AbD Serotec; RRID: AB_20745), Perilipin-1 (clone D1D8, Cell Signaling, RRID: AB_10829911), donkey anti-mouse IgG H&L (Alexa Fluor 488, Abcam, RRID: AB_2571721) and donkey anti-rabbit IgG H&L (Alexa Fluor 594, Abcam, RRID: AB_2734146). The slides were counterstained and mounted with a Vector TrueVIEW Autofluorescence Quenching Kit with DAPI (Vector laboratories).

After whole-slide image acquisition at 20x magnification (Aperio AT2, Leica Biosystems), semiquantitative measurements were performed via the VIS software platform (Visiopharm, Denmark) and the whole-slide image analysis platform HALO (Indica Labs, UK). Briefly, the workflow allows the quantification of the following cell densities across a given surface: a whole tissue section or fatty region, a 500 µm-wide rim area around the fatty region, and a tumor core. For manual delination of regions, each surface of adipose tissue (containing at least 10 adipocytes) was considered a VAT region, and a 500 µm-wide rim was measured and drawn around each VAT region. Each image was visually reviewed for quality control. The results are expressed as positive cells per square millimeter (mm^2^) in the region of interest.

For immunofluorescence staining, sections or cells were fixed with acetone:methanol (1:2 v/v) or paraformaldehyde (4%) and then permeabilized with 0.1% Triton X before incubation with the primary antibody and BODIPY 493/503 (Thermo Fisher). The secondary antibody used was Alexa Fluor 555-conjugated goat anti-mouse (Invitrogen), followed by DAPI nuclear staining. Fluorescent sections were scanned via a Nanozoomer S60 scanner (Hamamatsu Photonics). Immunocytofluorescence images were acquired via a BZ-X700 microscope (Keyence). Confocal imaging was performed at the Nikon Imaging Center (Heidelberg, Germany) on a C2 Plus confocal microscope (Nikon) at 20x magnification.

### Macrophage isolation from ascites and cell culture

Macrophages were isolated from ascites originating from patients with ovarian, breast, gastric, or cancer of unknown primary origin. The experiments were approved by the local ethics committee (S-22/2013), and informed consent was obtained from each patient.

After centrifugation of the ascites fluid to pellet the total cells, the macrophages were isolated via differential adherence (the nonadherent cells were removed after 30 min). Cell purity was systematically checked by CD163 immunohistochemistry, and a purity above 95% was considered appropriate. The cells were cultured in original ascites supernatant/RPMI medium supplemented with GlutaMAX 50/50 v/v. When the nonadherent cell fraction contained high amounts of viable tumor cells, the isolated macrophages were referred to as cancer-educated, as they co-existed with cancer cells for however long the ascites built up prior culture.

Treatments were applied to cells directly at the time of seeding in an 8-well chamber culture (Ibidi) or in a culture plate. The treatments included maraviroc (5 nM, Thermo Fisher), anti-CD36 (5 µg/ml, clone JC63.1, Abcam), entinostat (10 µM, Biozol), RGFP966 (10 µM, Selleckchem), ferrostatin-1 (1 µM, Selleckchem), liproxstatin-1 (1 µM, Selleckchem) or heat-killed *Bacteroides vulgatus* (10^7^ cfu/ml, see supplemental materials). After 18 h, the supernatants were collected, and the cells were washed and frozen for subsequent immunocytochemistry, phosphoprotein analysis or lipid peroxidation assays. For lipid peroxidation assays, one million cells were treated for 18 h prior to lysis and assay following the manufacturer’s instructions (Lipid Peroxidation (MDA) Assay Kit, Colorimetric, Abcam).

For fat-free culture of macrophages, the ascites supernatant was skimmed with CleanAscite (Biotech Support Group, USA) following the manufacturer’s recommendations.

### Reagents and treatments for cell and tissue culture

The cells or tissues were treated with maraviroc (5 nM, Sigma Aldrich, Germany), anti-CD36 (Abcam clone JC63.1, 2 µg/ml, RRID: AB_447608), SSO (100 µM, Cayman 11211), anti-CXCL9 (R&D AF392, 1 µg/ml, RRID: AB_355342), anti-CXCL10 (R&D AF266, 1 µg/ml, RRID: AB_354433), palmitic acid (200 µM), poly(I:C) (Sigma P0913, 20 µM), clodronate, or PBS liposomes (Liposoma, 1:10).

### Data from the UMIN000005714 trial

H&E slides from 20 patients from the UMIN000005714 trial were generated and kindly provided by Hamanishi et al. Virtual slide images were assessed for the presence of adipose tissue by two observers blinded to the clinical outcomes.

### Image-enabled cell sorting experiments

Image-enabled cell sorting (ICS) was performed via the ICS prototype described previously.^62^ The cells were prepared for ICS by staining with BODIPY 493/503 (0.2 mg/ml for 30 min) in the adherent culture prior to cell scraping and preparation of a single-cell suspension in PBS. For sorting, singlets were first isolated via forward/sideward scatter signals. Then, cells with very high or low fluorescence in the BODIPY channel were gated out via signal height and area parameters, and traffic/storage phenotypes were enriched via the maximum intensity signal of the BODIPY channel. Approximately 1400 cells were sorted in purity mode into 96-well glass bottom microscopy dishes. The cells at the bottom of the dish were collected via short centrifugation (90 × *g* for 20 s) to visually inspect the collected cells under a widefield microscope for blind validation of the ICS. For a more detailed description of the general ICS workflow and parameters, we refer to Schraivogel et al. Science 2022.^62^

### RNA isolation, RNA-sequencing and RNA-sequencing data analysis

Sorted cells were treated with maraviroc or anti-CD36 for 18 h before being subjected to RNA lysis in plates supplemented with 0.4% Triton X, RNase inhibitor, dNTPs, and oligodTs. Transcriptome libraries were prepared with the SMART-seq v3 Ultra Low Input RNA Kit (Takara) and sequenced via MiSeq (300 nts PE) at the Genomics Core Facility, EMBL Heidelberg.

Differential expression RNA analysis was carried out on the basis of the negative binomial distribution via the DESeq2 package version 1.40.2,^63^ and the log2 change values were decreased via the “apeglm” method.^64^ Differentially expressed genes (DEGs) with *p* < 0.05 and absolute fold2 change (FC) values ≥ 0.5 were identified. Gene set analysis via the Molecular Signatures Database (MsigDB) was performed via the packages fgsea (1.26.0) and msigdbr (7.5.1). The normalized enrichment score (NES) was plotted. All plots were generated via the ggplot2 (v. 3.4.4) and VennDiagramm (v. 1.7.3) packages in R 4.3.1, and p values are indicated.

### MISTRG mouse humanization

Experimental procedures were approved by the Institutional Animal Care and Use Committee and the local animal welfare committee of the Biopole ULB Charleroi (BUC) (int. ref. 40GosBuc). Within 48 h after birth, the pups were sublethally irradiated with a unique dose of 80 cGy (Gammacell 40 exactor MDS Nordion) and subsequently injected intrahepatically with 10,000 fetal liver-derived CD34+ cells via a 22 gauge needle (Hamilton Company). Six weeks post-injection, the level of human immune reconstitution in the blood was measured via flow cytometry, and the mice were considered humanized when the percentage of hCD45+ cells among total CD45+ cells (mCD45+ and hCD45+ cells) reached a minimum of 2% at 6 weeks.

### Tumor inoculation, treatment and monitoring

Tumors were initiated by orthotopic injection of 5 × 10^6^ MDA-MB-231 tumor cells (in 100 μL of sterile Matrigel, Corning 356234) per mouse into the mammary fat pad on day 0. On day 21, 7 mice were administered Maraviroc (Thermo Fisher Scientific), which was dissolved in the drinking water at a concentration of 300 mg/l, for 10 days. Fresh drinking bottles were prepared every 3 days. The mice were monitored every other day for tumor growth via fine calipers. The tumor volume (mm3) is described as (A × B2)/2, where A and B represent the tumor length and width, respectively. The mice were sacrificed for tumor analysis on day 31.

### Tissue digestion and leukocyte sorting

Tumors were dissected, finely chopped, and dissociated with a digestion solution containing 20 μL of 1 mg/mL DNAse I (Grade II, MilliporeSigma, 10104159001) and a mixture of 2.5 mg/mL collagenase I and II (Liberase TL Research Grade, Roche) in 5 mL of RPMI 1640 (Lonza), each of which was incubated for 40 min at 37 °C. After 5 mL of 5% RPMI FCS with 2 mM ethylenediaminetetraacetic acid (EDTA, Millipore Sigma) was added to each sample, the tumor pieces were mashed and filtered (100 µm). Mononuclear cells were isolated from dissociated tissues via Lymphoprep gradient centrifugation, followed by a washing step with 5% RPMI FCS with 2 mM EDTA. The cell suspensions were washed in PBS and incubated (for 30 min at 37 °C in the dark) in 50 μL of PBS containing an Fc-blocking reagent mixture (rat anti–mouse CD16/CD32, BD, clone 2.4G2, dilution 1/100 and human Fc block, BD) and incubated for 10 min at 4 °C. Cell surface staining was performed using monoclonal antibodies against the following molecules (clone, company): hCD45 APC Cy (BD, clone 2D1, mCD45 FITC (BD, clone 30-F-11), hCD33 APC (BD, clone WM53), hCD14 PB (BD, clone M5E2), and the LIVE/DEAD™ Fixable Aqua Dead Cell Stain Kit for 405 nm excitation (Invitrogen). Hashing antibodies (0.5 µg per 2 million cells, TotalSeq™-B anti-human Hashtag Antibodies, clones LNH-94 and 2M2; Biolegend) were added to the cell suspensions to discriminate individual mice. The cell suspensions were washed with 2% FCS in PBS, resuspended with 5% FPMI and filtered with a 40 µm filter prior to cell sorting. hCD45+ and mCD45+ living cells were isolated via a BD FACSAria III and then collected in a FBS precoated tube containing 300 µL of PBS + 20% FBS.

### Single-cell RNA library preparation, sequencing and alignment

For FACS sorting, live human CD45+ cells isolated from MDA-MB-231 tumor cell suspensions were filtered through a 40 μm cell strainer, and the concentration was adjusted to 1000 cells/μL before single-cell capture via a Chromium controller (10x Genomics). In total, cells from 7 untreated mice and 7 maraviroc-treated mice were sequenced. Single-cell RNA-seq libraries were prepared via the Chromium Single Cell 3’ v3.1 Reagent Kit (10x Genomics) according to the manufacturer’s instructions. Libraries were loaded onto an Illumina NovaSeq (Brightcore platform), and Cell Ranger 9.0.1 (10x Genomics) function count was used to demultiplex the sequencing data, generate gene‒barcode matrices, and align the sequences to the GRCh38 genome. The resulting matrices were used to generate a single-cell seurat object via the Seurat 5.1.0 R package.

#### Demultiplexing

Data containing information about oligo-tagged antibodies and cell barcodes were added as an independent assay to the Seurat object and normalized with the NormalizeData() function from the Seurat package (version 5.1.0) via the “CLR” normalization method, which applies a centered log ratio transformation. The HTODemux() function was used to reassign individual cells to their original mouse sample. Negative cells with no oligo-tagged antibody detected and cells with more than one oligo-tagged antibody were removed from downstream analysis.

#### Filtering

RNA data were filtered by mouse sample with the following thresholds: number of genes expressed > 600 (between 600 and 6000 depending on the mouse sample) and fraction of mitochondrial content < 12.5. The new data from the filtered Seurat object were normalized with NormalizeData () and scaled with ScaleData() functions from the Seurat package. The following variables were used for regression: ncount_RNA and the mitochondrial and ribosomal contents.

To remove batch effects and compare clusters between untreated and maraviroc-treated mice, we used the IntegrateLayers function with the method HarmonyIntegration from the Seurat package.

Variable features were used in the top 30 principal components (PCs) with the RunUMAP() function to identify clusters via the original Louvain algorithm with a resolution of 0.8.

The FindAllMarkers() function with default parameters was used to annotate major cell populations.

All myeloid cells were separated from CD45+ cells, reclustered with the same parameters and projected to a new UMAP with the same number of dimensions. The myeloid clusters were annotated on the basis of the differentially expressed genes determined via the FindAllMarkers() function.

### Statistical analysis

Measured values with a normal distribution were compared via two-tailed Student’s *t* test or two-way ANOVA in the case of multiple comparisons.

For the results of the in vitro experiments that did not have a normal distribution, the measured values were compared via Wilcoxon’s rank test (for paired analysis) or the Mann‒Whitney *U* test. Dunn’s multiple comparison test was applied for multiple comparisons. The sample size and statistical test results are shown in the figure legends. Statistical *P* value was set at *P* < 0.05. Abbreviations: not significant (ns) = *P* > 0.05; *P < 0.05; **P < 0.01; ***P < 0.001. For the results displayed with whisker plots, the plots represent the median, 1.5*IQR, min and max values.

## Data and materials availability

The scRNA-sequencing data from humanized mice have been deposited in the Gene Expression Omnibus (GEO) repository and are available at https://www.ncbi.nlm.nih.gov/geo under accession number GSE310918. The requests for further information and resources should be directed to and will be fulfilled by the lead contact.

## Supplementary information

Revised_Supplementary Materials and FIgures_mark up

## Abbreviations

AKT/PKB: protein kinase B
CCL2: C-C motif chemokine ligand 2
C1Q: complement component 1q
GLUT1: glucose transporter 1
GSK-3β: glycogen synthase kinase 3 beta
HMOX1: heme oxygenase 1
IFN-γ: interferon gamma
IL-1β: interleukin 1 beta
IL-6: interleukin 6
IL-8: interleukin 8
MAF: musculoaponeurotic fibrosarcoma oncogene homolog
MoMac-VERSE: Monocytes macrophages VERSE
MT1G: metallothionein 1G
SPP1: secreted phosphoprotein 1
STAT3: signal transducer and activator of transcription 3
TNF-α: tumor necrosis factor alpha
TREM2: triggering receptor expressed on myeloid cells 2
