# Supplementary material for: Harnessing lipid-driven immunometabolic pathways in omental metastases to enhance immunotherapy in patients with ovarian cancer
Source: Signal Transduct Target Ther. 2026 Mar 4;11:78. doi: 10.1038/s41392-026-02594-8 (PMC12957430; doi:10.1038/s41392-026-02594-8)
Supplement: Supplementary file 1 — Revised_Supplementary Materials and FIgures_mark up [file 41392_2026_2594_MOESM1_ESM.docx]

Supplementary Materials for

Harnessing lipid-driven immuno-metabolic pathways in omental metastases to enhance immunotherapy in ovarian cancer

**Authors:** Meggy Suarez-Carmona^1,2,3^, Mareike Hampel^2^, Xin-Wen Zhang^1, 2, 3^, Alexandra Pöchmann^1,3,4^, Silke A. Grauling-Halama^1,3^, Nektarios A. Valous^5, 6^, Pornpimol Charoentong^2,5, 6^, Dyke Ferber^2^, Jannis Wissfeld^3,7^, Alicia Höflich^1,3^, Stanislas Goriely^8,9^, Aurélie Detavernier^8,9^, Abdulkader Azouz^8,9^, Anthony Rongvaux^10^, Sven Zukunft^11,12^, Ingrid Fleming^11,12^, Jürgen G. Okun^13^, Vickie Baracos^14^, Mathias Heikenwalder^15^, Laurence Zitvogel^16^, Xinyi Xu^17^, Chenqi Xu^17^, Michael Volkmar^18^, Daniel Schraivogel^19^, Lars Steinmetz^19^, Junzo Hamanishi^20^, Masaki Mandai^20^, Mathias Gaida^21-22^, Theresa Mokry^23-24^, Johanna Nattenmüller^23-25^, Oliver Sedlaczek^23-24^, Nanna Monje^27^, Roxana Schwab^27^, Annette Hasenburg Athanasios Mavratzas^26^, Regina Johanna Boger^26^, Frederik Marmé^26^, , Sarah Schott^27^, Niels Halama^1,2,3,28,,29^*

Correspondence to: [niels.halama@dkfz-heidelberg.de](mailto:niels.halama@dkfz-heidelberg.de)

**This PDF file includes:**

Materials and Methods

Methods References

Supplementary Figures. 1 to 11

Supplementary Table 1

Materials and Methods

Histology: virtual overlay

The computational workflow for creating the three-color virtual overlay was based on linear image registration and biomarker segmentation of sequential immunostained sections (4 μm). Initially, background from the immunostained slides (CA125, CD3+, and CD8+) was removed using 2D histogram variance thresholding ^1^. Then, rigid (translation and rotation) registration was carried out using the CD3 immunostain image as the target in the original scale. Finally, biomarker segmentation was carried out on the aligned imagery involved stain separation using color deconvolution ^2^ and post-processing operations ^3^. The resulting binary images were merged into a virtual section and rendered in pseudocolor (red for CA125, green for CD3+, and blue for CD8).

Multiplex cytokine and phosphoprotein analysis

Multiplex cytokine quantification was performed on cell culture supernatants and tissue lysates as previously described ^31^. Briefly, sections of cryopreserved tissue were collected and lysed using the Bio-Plex cell lysis kit (Bio-Rad) and the lysate concentration was adjusted to 300µg/ml. The protein concentration of soluble factors was determined using the Bio-Plex Pro^TM^ human cytokine assays, allowing the absolute measurement of fifty different soluble factors (Bio-Plex Pro^TM^ human cytokine, chemokine, and growth factor assay, Bio-Rad) according to the manufacturer's instructions. The factors are: IL-1b, IL-1ra, IL-2, IL-4, IL-5, IL-6, IL-7, IL-8, IL-9, IL-10, IL-12p70, IL-13, IL-15, IL-17, Eotaxin, FGFb, G-CSF, GM-CSF, IFN-g, CXCL10, CCL2/MCP-1, CCL3/MIP-1a, CCL4/MIP-1b, PDGFbb, CCL5, TNF-a, VEGF, CCL27, GROa, HGF, ICAM-1, IFN-a2, IL-2Ra, IL-3, IL-12p40, IL-16, IL-18, LIF, CCL7, M-CSF, MIF, CXCL9, b-NGF, SCF, SCGF, CXCL12, TNF-b, TRAIL, VCAM-1 and IL-1a. For phosphoprotein analysis, Phosphoplex analyses were performed according to the manufacturer’s instructions (BioRad, USA) and relative changes were analyzed. Bio-Plex Pro Cell Signaling MAPK Panel 9-plex, Bio-Plex Pro Cell Signaling Akt Panel 8-plex were used for phosphorylated protein quantification and Bio-Plex Pro Total Akt, Bio-Plex Pro Total ERK1/2 , Bio-Plex Pro Total GSK-3β, Bio-Plex Pro Total JNK, Bio-Plex Pro Total MEK1, Bio-Plex Pro Total PTEN , Bio-Plex Pro Total mTOR , Bio-Plex Pro Total p38 MAPK, Bio-Plex Pro Total p70 S6 Kinase, Bio-Plex Pro Total Human GAPDH, Bio-Plex Pro Total β-Actin were used for quantification of total proteins.

Isolation of tumor-infiltrating cytotoxic T cells and tumor cells, cytotoxicity assay

To study expression profiles of genes involved in cholesterol metabolism, CD8+ T cells were isolated from fresh tumors using CD8 magnetic beads (Miltenyi Biotec) after tumor dissociation (tumor dissociation kit, Miltenyi Biotec) following the manufacturer’s instructions.

For autologous cytotoxicity assay, TILs and tumors cells were isolated from dissociated omentum metastases. Briefly, tissue was dissociated using tumor dissociation kit (Miltenyi) following the manufacturer’s instruction. Dissociated tissue was used for CD3+ T cell isolation using EasySep™ Release Human CD3 Positive Selection Kit (Stemcell Technologies) and the T cells were cultured in X-VIVO15 medium supplemented with 2% human serumalbumin, penicillin-streptomycin (Gibco) and recombinant IL-2 (6000 U/ml). The remainder was used for tumor cell isolation by adherence. Cancer cells were left to adhere overnight in RPMI-10% FCS supplemented with penicillin-streptomycin, non essential amino acids, sodium pyruvate and Glutamax (all from Gibo) and were used at passages 2 to 5. TIL and cancer cells purity were evaluated by immunohistochemistry for CD3 and CA125 on a cytospin. Cytotoxicity assays were carried out using the CYQUANT LDH cytotoxicity assay (Invitrogen) according to the manufacturer’s instructions, including a prior measurement of cancer cells viability. Cancer cells were seeded in 96-well plates and left to adhere overnight. TILs were added in their medium to yield various effector to target ratios and the co-culture was incubated for 48 hours, prior to analysis of the LDH concentration in the supernatant. Cytotoxicity was evaluated as follows:

$$\% cytotoxicity= \frac{\left( experimental value-spontaneous release control \right)}{\left( maximum release control-spontaneous release control \right)} x 100$$

Proliferation assay of naïve T cells

Naïve T cells were isolated from PBMCs with the EasySep™ Release Human CD3 Positive Selection Kit (StemCell) according to the manufacturer’s instruction and T cell purity was confirmed by CD3 immunostaining. T cells were suspended at a concentration of 1 million cells per ml and stained with CMFDA (Invitrogen, 0.5 µM in PBS-1% human serum albumin), generating a bright signal. T cells were cultured in RPMI supplemented with 10% FBS, Interleukin-2 (50 U/ml), beta-mercaptoethanol (0.01 mM), penicillin-streptomycin (100 U/ml) and treated either with recombinant human CXCL9 and CXCL10 (Peprotech) or with 50 % conditioned medium from EOC. After 7 days, T cells were analyzed by flow cytometry (Cytek Aurora).

Cholesterol metabolism-related gene expression

RNA was extracted using the RNeasy mini kit (Qiagen) and reverse transcribed into cDNA (Maxima H Minus First Strand cDNA synthesis kit, ThermoFisher). The PCR was conducted with the primers referenced previously ^27^.

Metabolomics profiling of TILs exposed to adipocyte supernatant

TILs were cultured either in culture medium (DMEM supplemented with 10% heat-inactivated fetal calf serum and 1% L-Glutamax), or in conditioned medium from adipocytes. For the generation of conditioned medium, adipocytes were isolated from healthy breast tissue surgically removed during breast reduction surgery. Tissue was washed three times in saline, then incubated in digestion medium (DMEM + 1% GlutaMAX + 4% BSA + 1mg/ml collagenase I) for 45 minutes at 37°C with constant gently agitation. Digestion was stopped by dilution with 1 volume culture medium, the tissue was filtered through a 100 µm mesh, centrifuged and resuspended in fresh culture medium. The conditioned medium was harvested after 48 hours and stored at -20°C until use.

TILs were cultured in conditioned medium for 72 hours, pelleted and washed in PBS. Metabolomics profiling with the Absolute IDQ p180 kit (Biocrates) was carried out following the manufacturer’s instructions.

Bacterial species cultivation and identification

Fresh tumor tissue specimens were minced under sterile conditions and cultured in thioglycolate broth containing hemin and vitamin K1 at 37°C and 5% CO2. After 48h broths were seeded on chocolate and Schaedler agar plates and cultivated for 8 weeks or until visible growth. Chocolate agar plates were incubated at 37°C under 5% CO_2_. Anaerobic culture on Schaedler agar plates was performed in anaerobic jars at 37°C.

Bacterial DNA was extracted with the RTP® Bacteria DNA Mini Kit (Stratec Molecular, Berlin) following the manufacturer’s instructions. Isolates were identified according to standard microbiology protocols. Upon noted bacterial growth (confirmed on agar plates), 16s sequencing of the 16S rRNA gene with the eubacterial primers S-D-Bact-0008-a-S-16 and S-D-Bact-1492-a-A-16 and sequence analysis was performed using the SILVA database as described previously to identify the cultured species. *Bacteroides vulgatus* was derived from a clinical sample and species was confirmed by routine mass spectrometry.

Adipose tissue quantification in CT data

Routine imaging data from magnetic resonance imaging was obtained, and corresponding BMI and evaluation of imaging studies was performed by a board-certified radiologist, blinded to the corresponding histology. Area-based quantification of adipose tissue by using CT data was performed with a semiautomatic volume tool (Syngo Multimodality Workplace, Siemens Healthcare, Munich, Berlin, Germany) at lumbar spinal level L3/4 ^4,5^. Specific regions of interest (ROI) for analysis of the total adipose tissue (TAT, whole abdominal circumference) and visceral adipose tissue (VAT, delineating the abdominal wall along the fascial plane and the vertebral body`s anterior edge) were performed (volumetric quantification of selected slice, divided by slice thickness). Adipose tissue was selected by using a lower attenuation limit of -190 HU and an upper attenuation limit of -30 HU ^5,6^. SFA was calculated by subtracting VFA from TFA. In this manuscript, MRI images are displayed in the figures for illustration purposes, though CT-scans were used for the analysis.

Classical machine learning and statistics

For imaging adipose tissue and histology correlations, datasets were compiled from SyngoMMWP data and histological analyses. From this input vector it is clear that it is a feature vector without spatial structure (areas, volumes, functional parameters of T cell expansion, adipose tissue localization to tumor, etc.) and the quantity of available datasets is limited. Therefore a classical machine learning approach utilizing decision trees was performed (R statistical computing language, version 4, rpart package, version 4.1-15). For wet lab experiment results, statistical analysis was performed on GraphPad Prism 8 (RRID: SCR_002798). Sample groups were compared using the non-parametric Mann-Whitney U test or Wilcoxon signed-rank test when appropriate (to compare paired samples). Fisher’s exact test was used for categorical comparisons. When comparing two groups with *n* > 35 for each set or when small groups displayed a normal distribution (Kolmogorov-Smirnov test of normality) and similar variances, parametric tests (paired t-tests) were used. Group variations of multiple factors were studied using two-way ANOVA with two-tailed *p*-value. Differences were considered statistically significant provided that the (two-tailed) *p*-value was less than 0.05. In histograms, the mean (± SEM) was plotted. *: *p*-value < 0.05; **: *p*-value < 0.01; ***: *p*-value < 0.001 and ****: *p*-value < 0.0001.

**
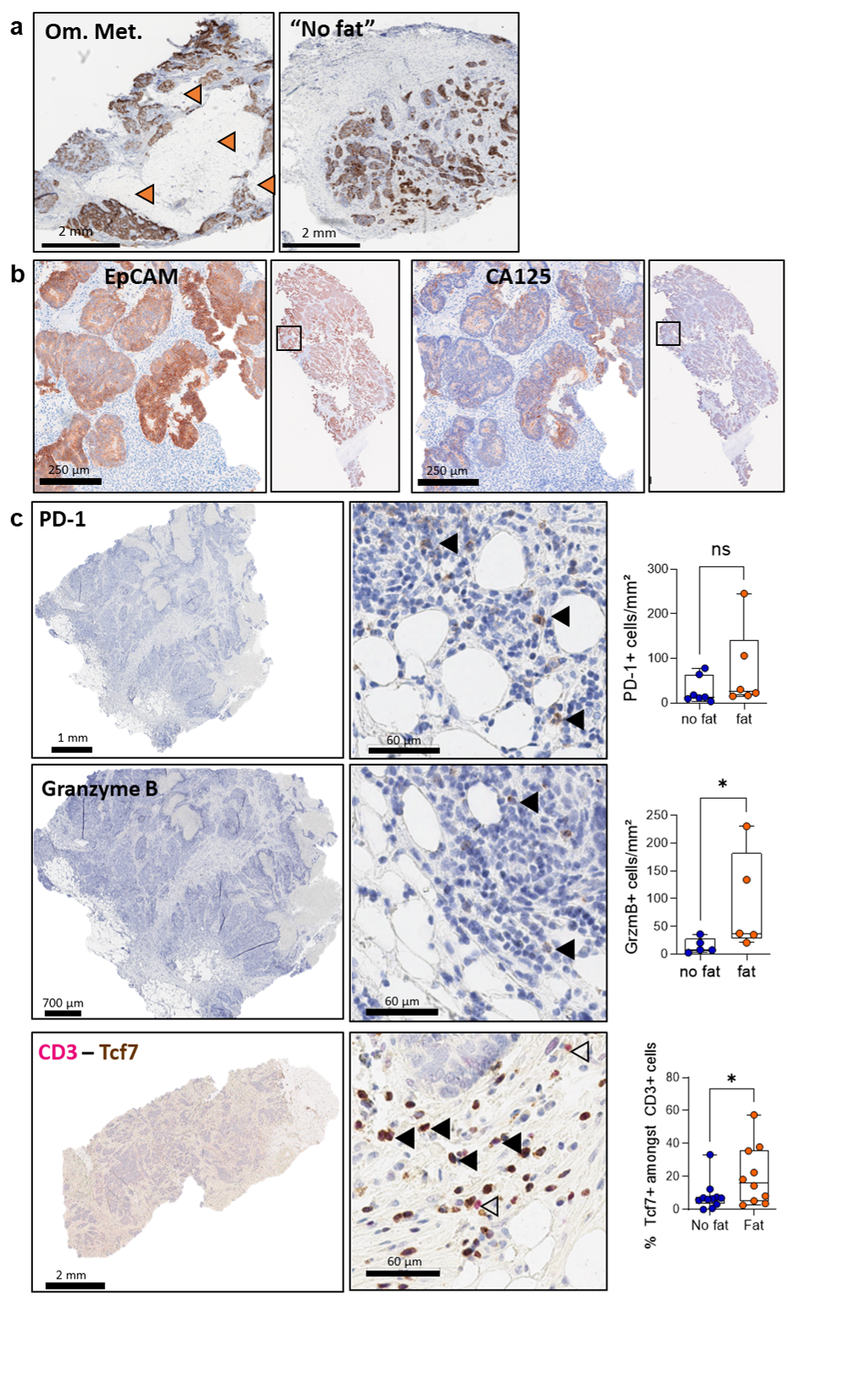
Supplementary Figure 1**

Supplementary Figure 1: Histological classification of EOC specimens in omentum metastases versus other sites. a Representative micro-images of CA125 immunostaining of an omentum metastasis and of an EOC specimen on another site, devoid of adipose tissue. Orange arrowheads point to adipose tissue areas. b Representative micro-images of a CA125 and an EpCAM immunostaining on serial sections of an EOC specimen, to detect tumor cells. c Representative micro-images of PD-1, Granzyme B and CD3-Tcf7 immunostainings on EOC sections as well as the corresponding quantification results on whole slide images from *n* = 10 (PD-1 and Granzyme B) or *n* = 20 EOC (CD3-Tcf7). Statistics: Mann Whitney test.


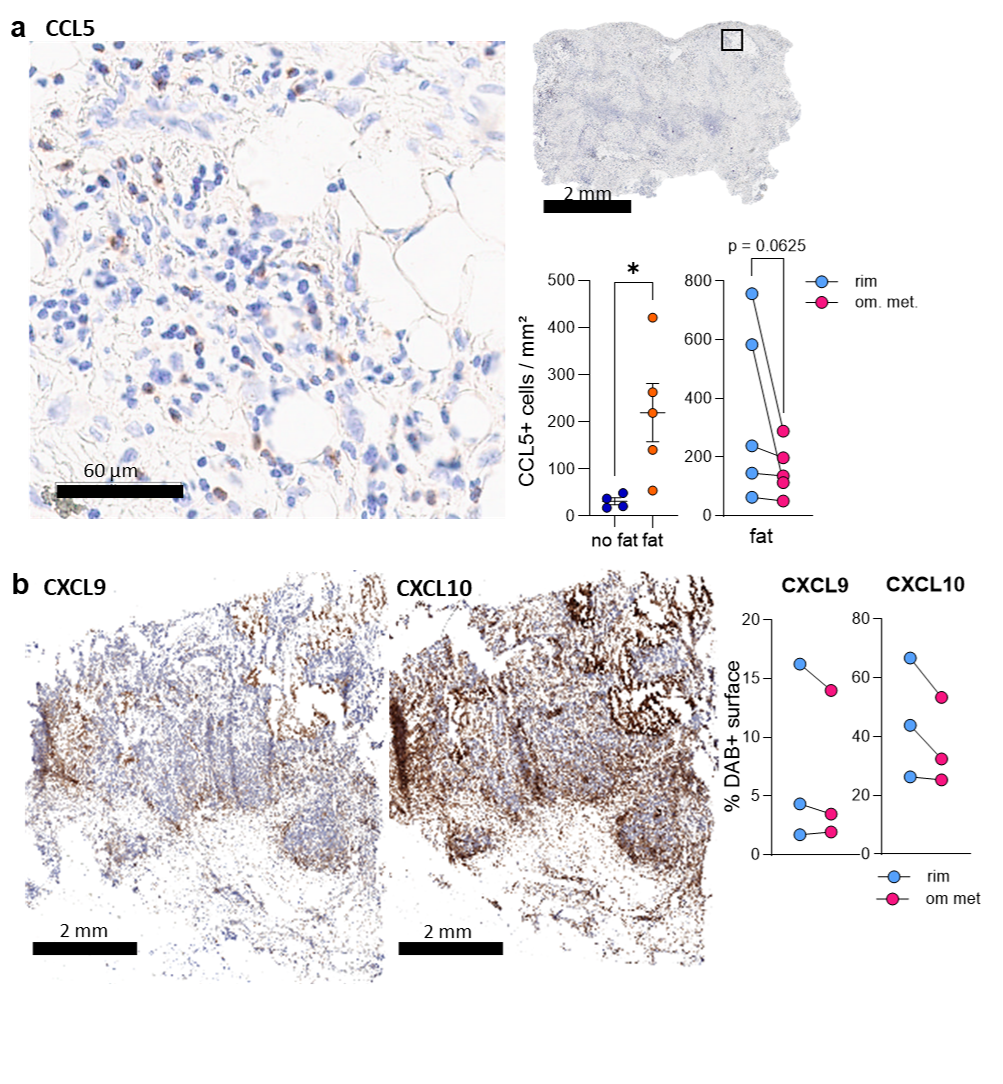
Supplementary Figure 2

Supplementary Figure 2: Spatial distribution of chemokines enriched in omentum metastases: CCL5, CXCL9 and CXCL10. a immunohistochemistry of CCL5: representative micro-images and corresponding quantifications in whole-slide analysis (n = 9 EOC specimens) and in spatially-define regions (n = 5 omentum metastases). b Immunohistochemistry for CXCL9 and CXCL10: representative micro-images and quantification of the proportion of the tissue surface occupied by DAB signal in QuPath (n = 3 or 4 omentum metastases).

Supplementary Figure 3

**
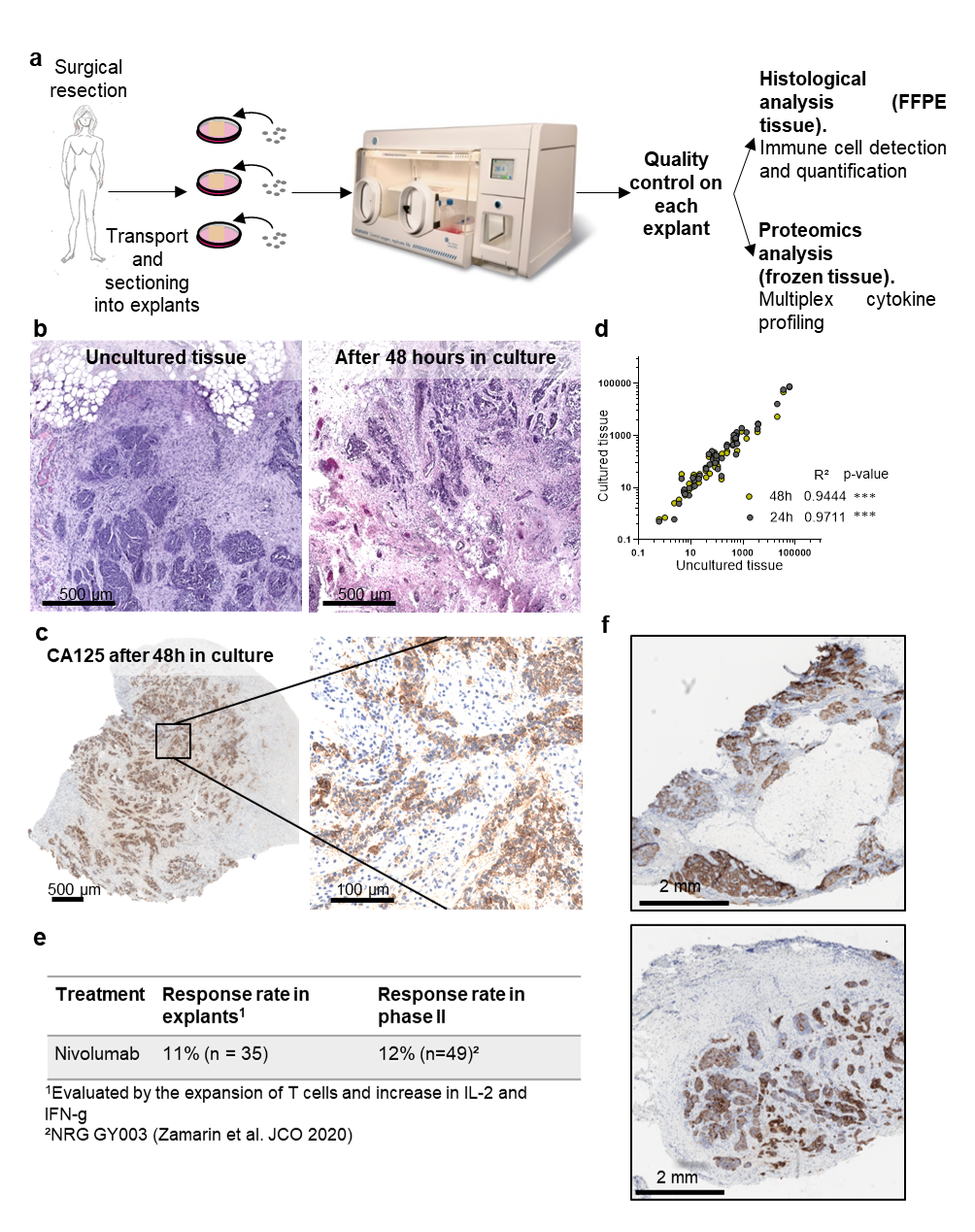
**

**Supplementary Figure 3: ovarian cancer tissue explant culture model and quality controls in primary tumors and metastases.** **a** Pipeline established for EOC explant culture and examples of successful quality control for tissue integrity, including **b** hematoxylin-eosin of a tissue before and after 48h of culture, **c** tumor cell content measured by CA125 immunostaining and **d** cytokine stability, assessed by spearman correlation plots comparing cultured and non-cultured tissues. **e** Response rates to Nivolumab in explant culture and in the clinics. **f** Representative micro-images of fat-containing tumors (omental metastases) and fat-free tumors (primary tumors).


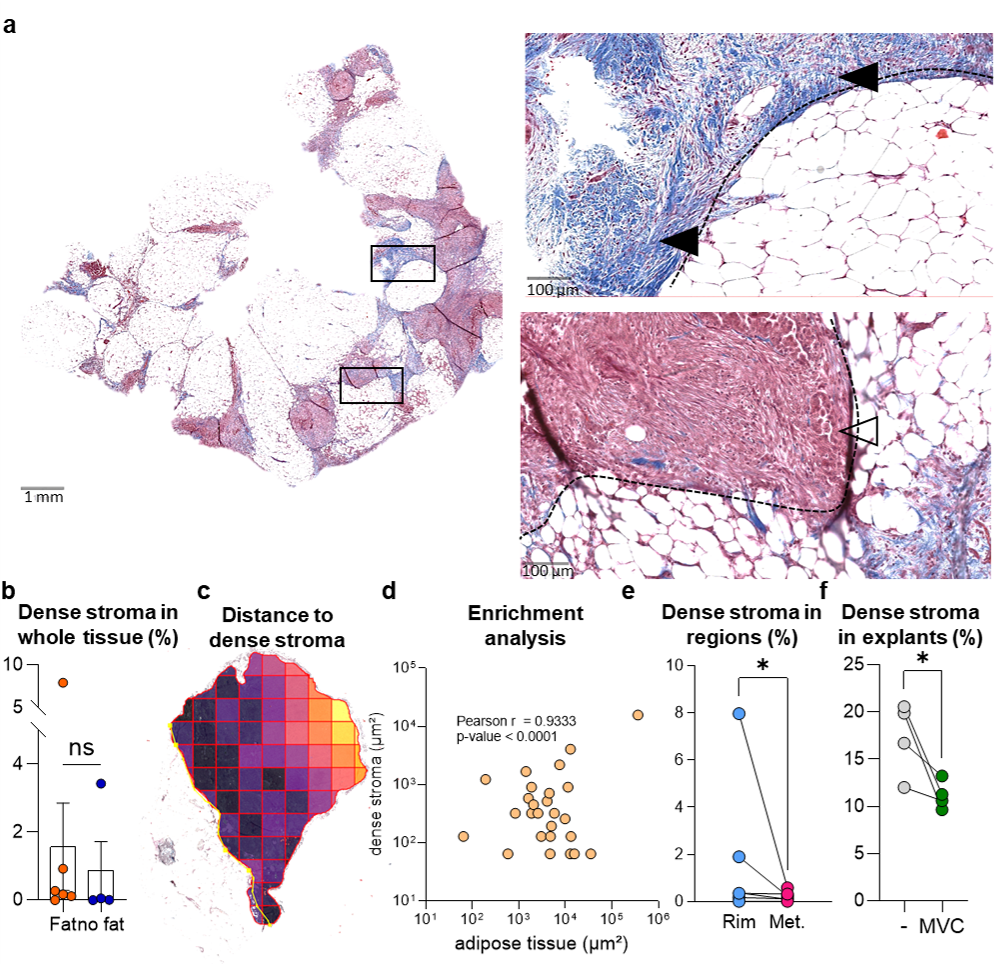
**Supplementary Figure 4**

**Supplementary Figure 4: Fibrotic compartment in omentum metastases is enriched in the omentum-metastasis interface and modulated by maraviroc. a** Illustrative micro-images of a Masson trichrome stain in a FFPE section of an EOC omentum metastasis specimen, depicting cytokeratin in red and collagen fibers in blue. Full black arrowheads indicate regions with direct proximity between collagen fibers and the omentum. Empty arrowheads indicate regions with direct contact between tumor cells and the omentum. **b** Proportion of EOC tissue surface occupied by dense stroma, in omentum metastases versus other sites (n = 10) **c** Illustrative image of the distance to dense stroma in an omentum metastasis, per 500x500 mm tile and **d** corresponding enrichment analysis. **e** Proportion of dense stroma surface in difference regions of omentum metastases (n = 5). **f** Proportion of dense stroma surface in explants from 4 EOC tissues cultured with maraviroc for 48 hours. Statistics: Wilcoxon signed rank test.

**Supplementary Figure 5**

**
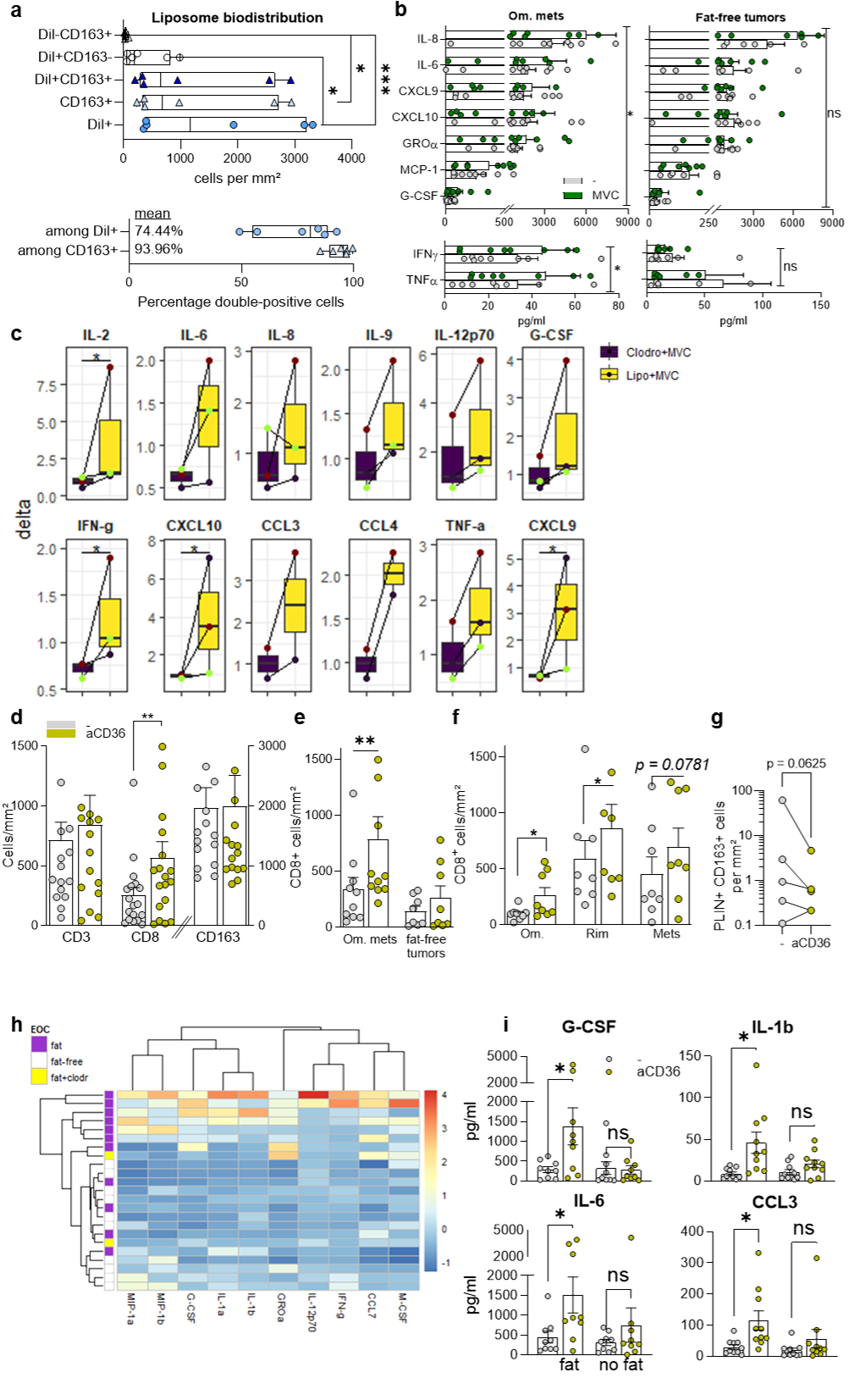
**

**Supplementary Figure 5: maraviroc and CD36 modulation in EOC tissues leads to CD8^+^ TIL expansion and cytokine remodeling**. **a** liposome biodistribution, expressed in cells per mm² (top) inr proportion of double positive cells for CD163 (for TAMs) and Dil liposomes (fluorescent liposomes). Each dot represent results from whole slide image analysis from an independent experiment. Statistics: Friedman Test. **b** Cytokine concentrations in whole tumor lysates from explants of omentum metastases or other sites treated with maraviroc. Statistical analysis: ANOVA. **c** differences (delta) in the cytokine changes induced by maraviroc in explants pre-treated with clodronate liposomes (clodro+MVC) or explants treated with negative control liposomes (lipo+MVC). Statistics: Wilcoxon ranks test. **d-e** Histological density of immune cells on whole slide images of EOC explants, cultured with a blocking anti-CD36 antibody. The density was measured **d** in whole tissue sections of all tissues (*n* = 18), **e** of fat-containing (*n* = 9) vs fat-free (*n* *= 9*) tissues, and **f** in manually delineated areas in omentum metastases (*n* = 8). **g** Number of perilipin-2+CD163+ TAMs per mm² in explants treated with anti-CD36 (n = 5). Statistics: Wilcoxon signed-rank test. **h-i** Selected cytokine changes in lysates of the corresponding EOC explants and in two omentum metastases pre-treated with clodronate liposomes (fat+clodr). Statistical tests: paired t test.

Supplementary Figure 6


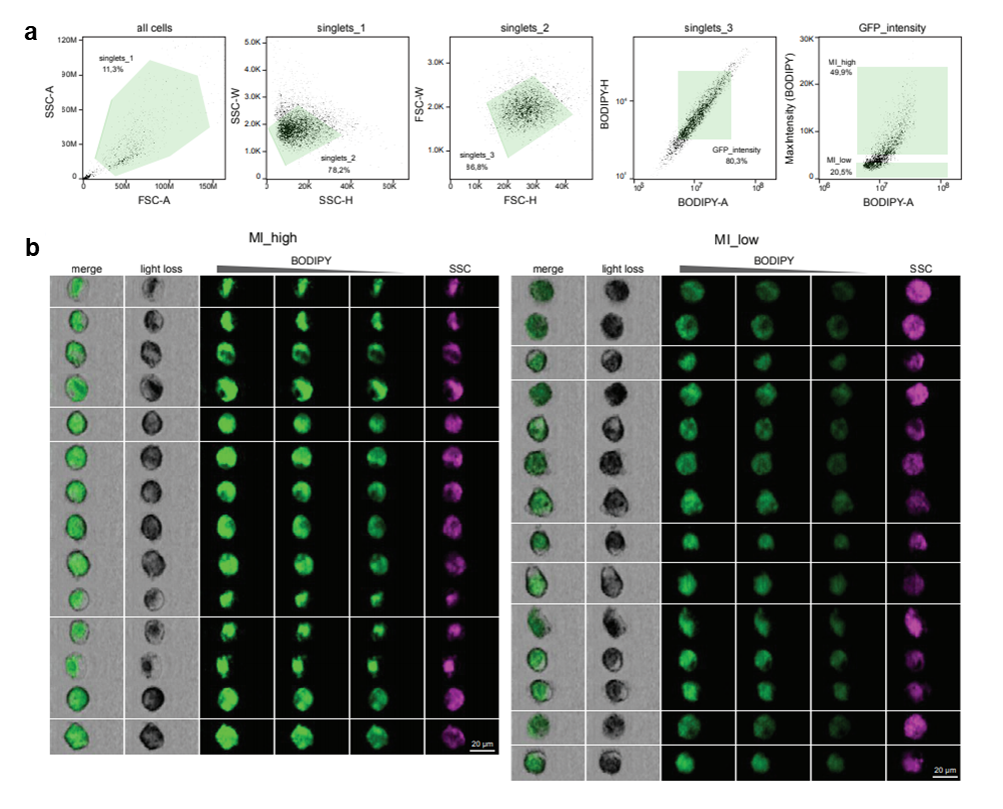
**Supplementary Figure 6: image-enabled cell sorting (ICS): visual parameters.** **a** Gating strategy for ICS. **b** Representative micro-images of MI^high^ and MI^low^ cells.


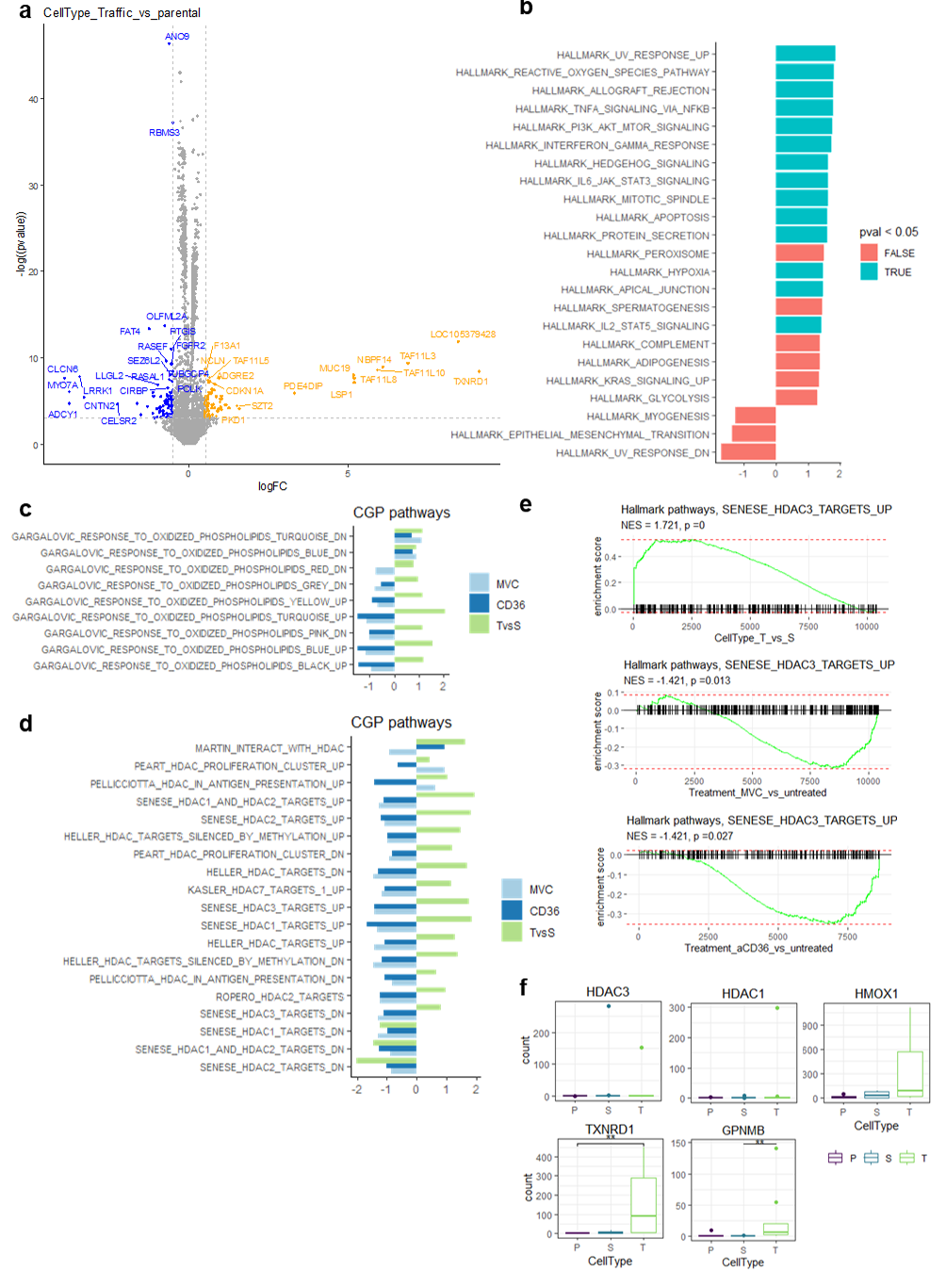
Supplementary Figure 7

**Supplementary Figure 7: RNA-seq analysis of sorted subpopulations of ascites-associated macrophages.** **a-b** Volcano plot highlight the top enriched or suppressed transcripts in (a) traffic cells compared to the parental population. **c** Gene set analysis of hallmark pathways differentially regulated between traffic and storage cells. **c-d** normalized GSEA scores in traffic versus storage cells (TvsS), or under maraviroc or anti-CD36 treatment, focusing on **c** response to oxidized phospholipids (from Gargalovic et al. (*50*)) or on **d** HDACs (from Senese et al. (*30*)). **e** Enrichment plots corresponding to the plots in **d** illustrating the enrichment in HDAC3 targets in TvsS, maraviroc- or anti-CD36-treated cells. **f** Individual expression of selected genes in sorted cells.

**Supplementary Figure 8**

**
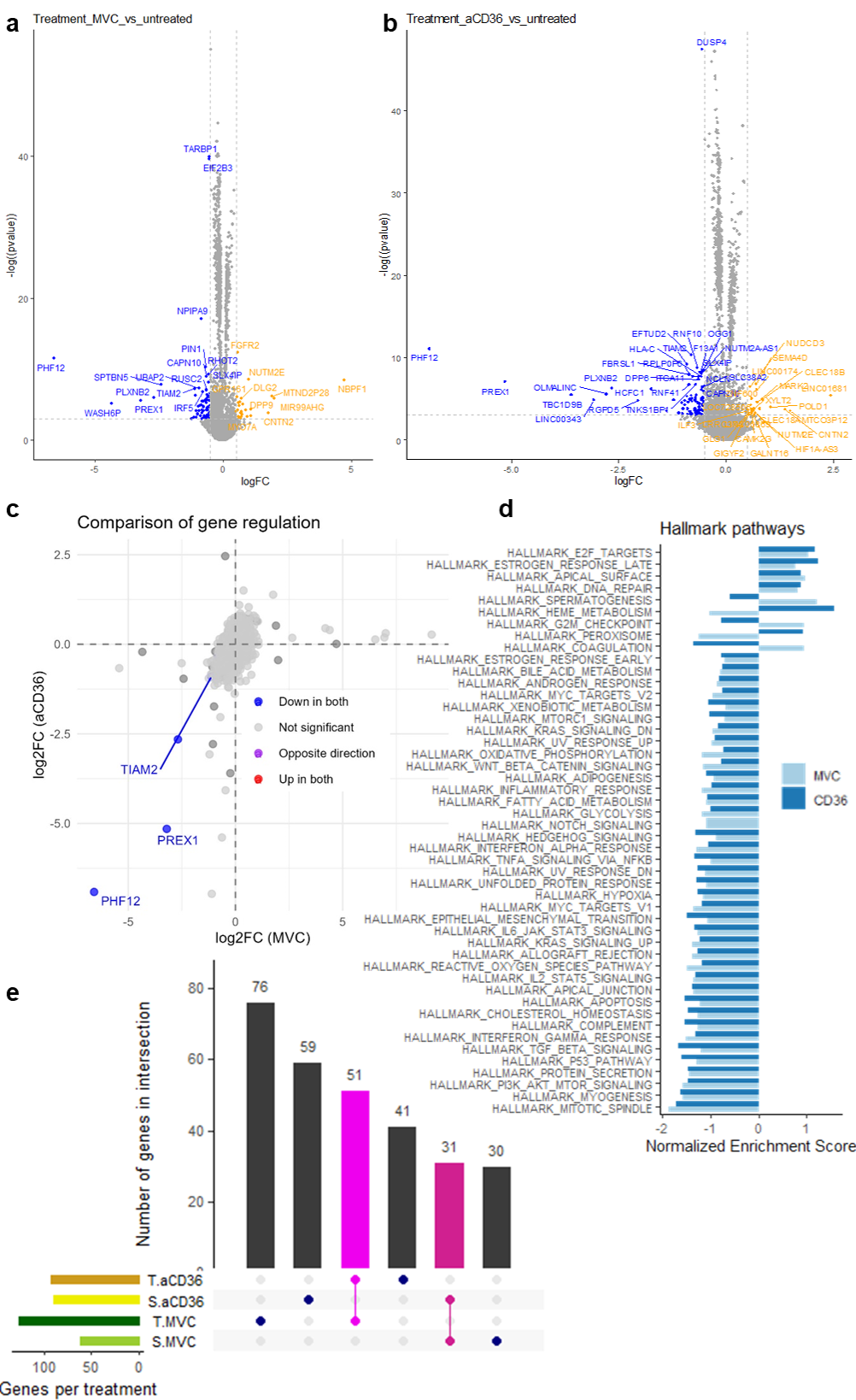
**

**Supplementary Figure 8: RNA-seq analysis of ascites-associated macrophages treated with maraviroc or anti-CD36. a-b** Volcano plots highlight the top modulated transcripts upon **a** maraviroc or **b** anti-CD36 treatment in all AAMs. **c** scatterplot of modulated genes illustrating the commonly upregulated or downregulated genes in maraviroc-treated cells and anti-CD36-treated cells **d** GSEA scores in Maraviroc- and anti-CD36-treated cells. **e** Upset plot illustrating the gene intersections between different populations and treatment.

**Supplementary Figure 9**


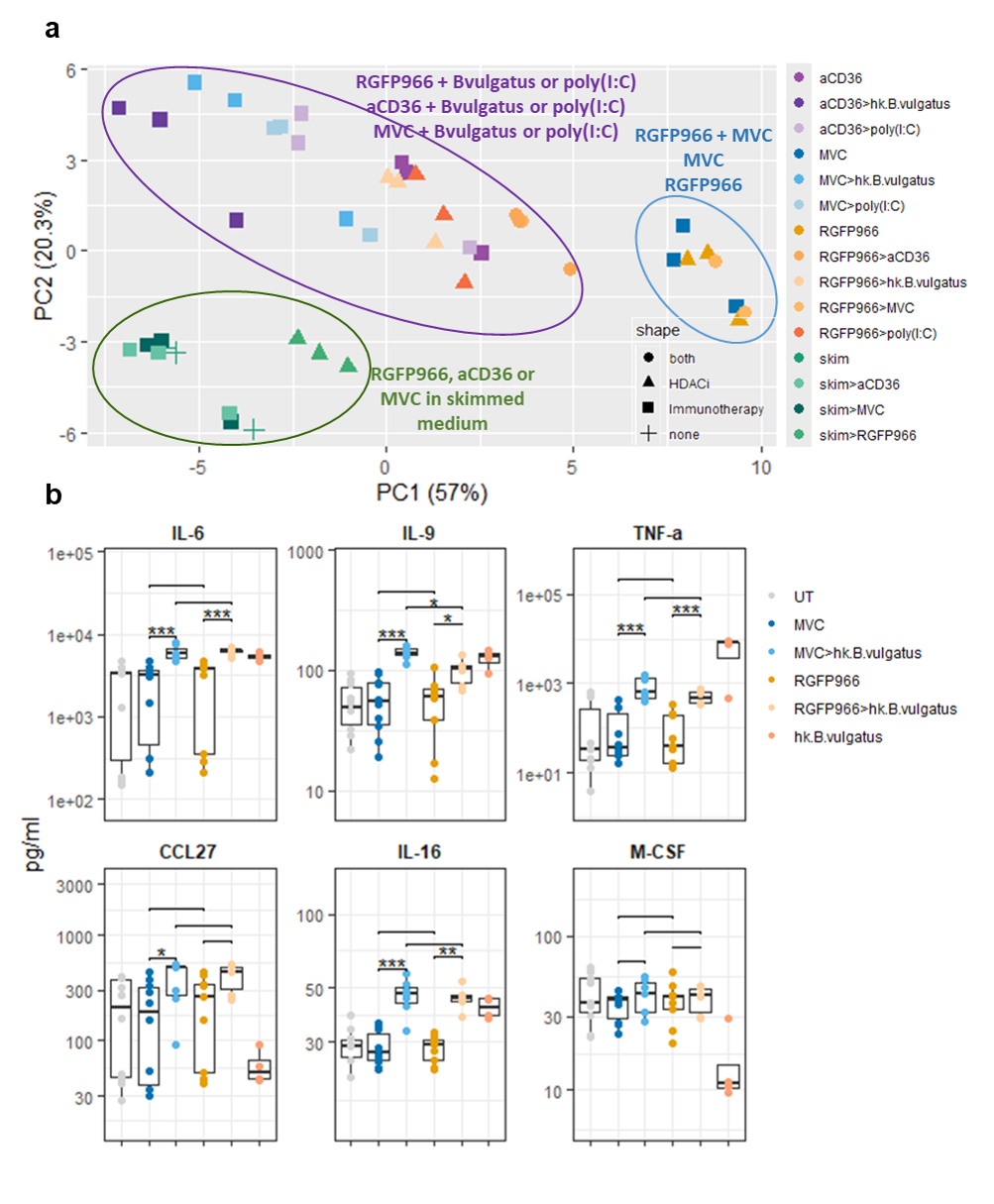


**Supplementary Figure 9: targeted proteomics analysis of AAMs treated with anti-CD36, maraviroc or RGFP91.** **a** Principal component analysis on the concentration of 47 soluble factors in the supernatant of AAMs after various treatments. The triplicate results of one representative experiment out of two are shown. **b** Concentration of selected soluble factors used for the PCA shown in **a** in the supernatant of AAMs. The results of two independent experiments in triplicates are plotted. Conditions are compared with a paired T test.

**Supplementary Figure 10
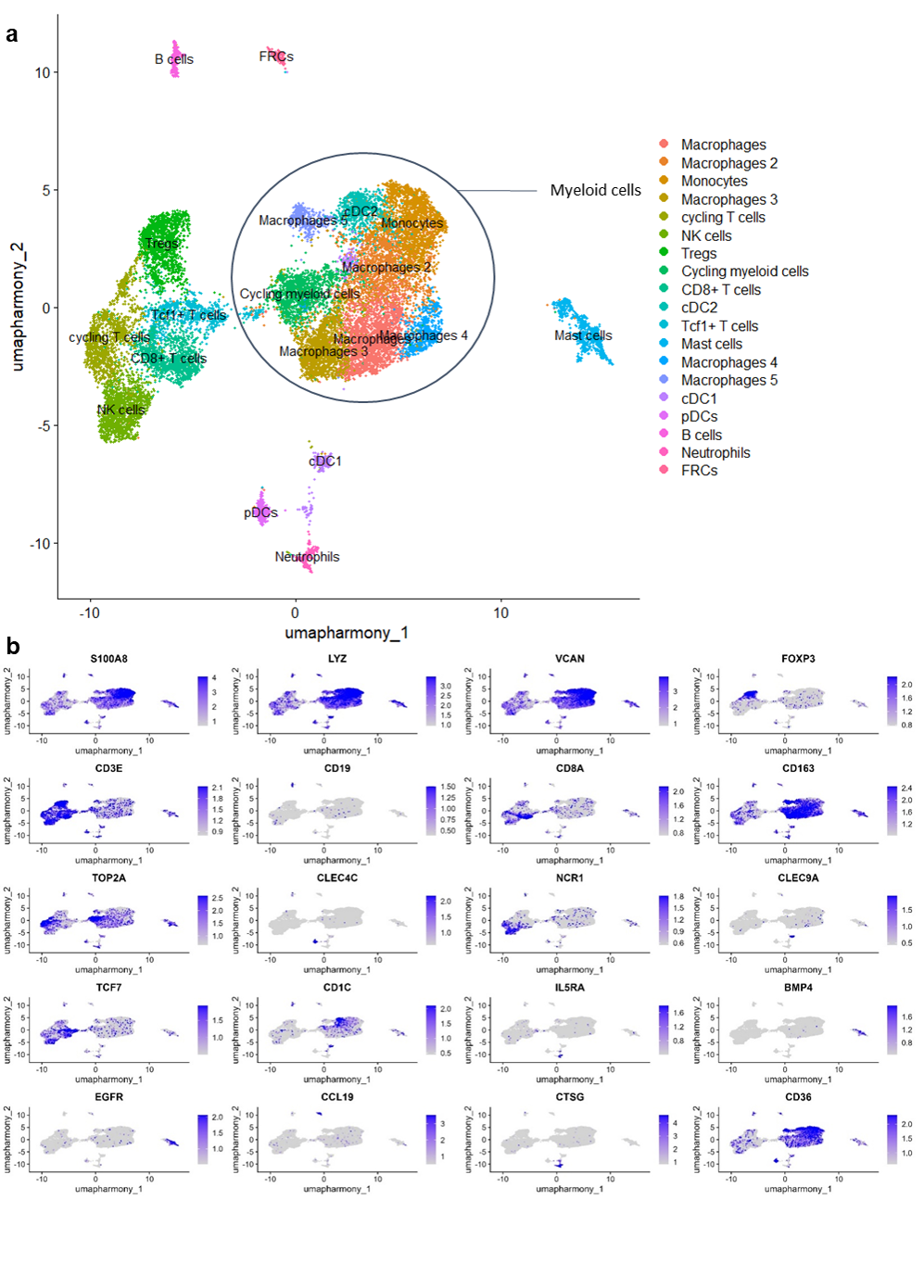
**

**Supplementary Figure 10: Infiltration of MDA-MB-231 tumors by lymphoid and myeloid cells in MISTRG mice.** **a** UMAP plots of tumor-infiltating human CD45+ cells in MISTRG mice, Each cluster is shown in a different color. **b** Feature plot of key marker gene expression.

**Supplementary Figure 11.**


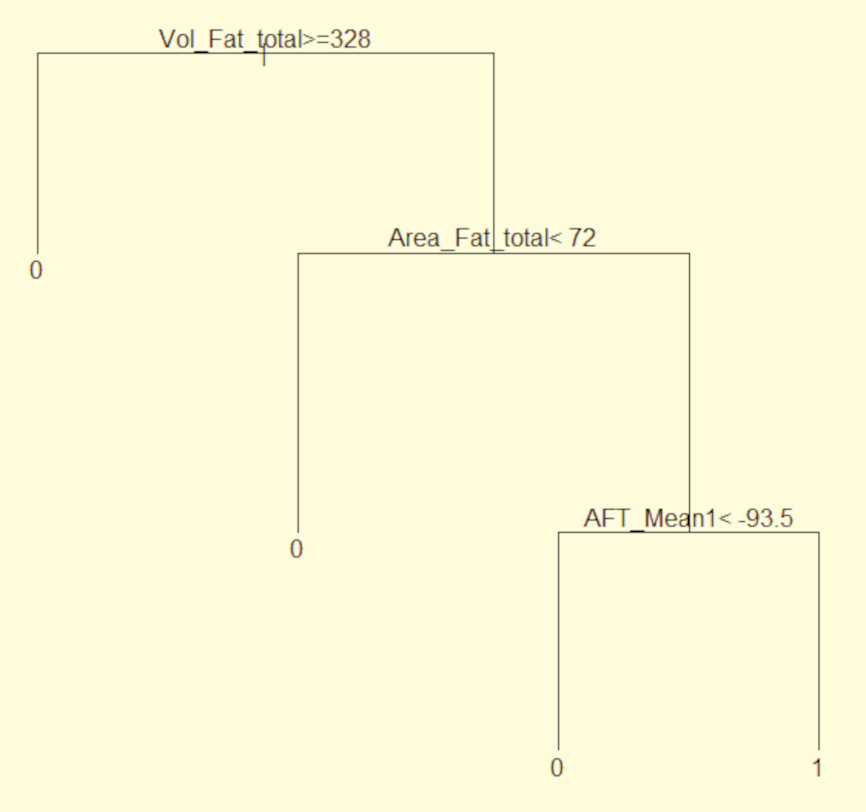


**Supplementary Figure 11: Recursive partitioning classifies patients’ MRI according to adipose tissue content and likely response to immunotherapy**. Recursive partitioning predicting response to immunotherapy (0 = profile irresponsive to immunotherapy and 1 = likely response to immunotherapy) using the visceral and total adipose tissue automatically detected on MRI.

Supplementary Table 1.

|  | No omentum infiltration | Omentum infiltration | Total |
| --- | --- | --- | --- |
| **Histological classification** | **50** | **54** | **104** |
| Serous adenocarcinoma | 35 | 44 | 79 |
| High-grade | 27 | 38 | 65 |
| Low-grade | 4 | 3 | 7 |
| Not graded | 4 | 3 | 7 |
| Mucinous adenocarcinoma | 3 | 1 | 4 |
| Endometrioid adenocarcinoma | 3 | 1 | 4 |
| Clear cell adenocarcinoma | 1 | 1 | 2 |
| Carcinoma, nfd | 2 | 5 | 7 |
| Granulosa cell tumor | 2 | 0 | 2 |
| Sarcoma | 1 | 0 | 1 |
| Unknown, CA125-positive | 3 | 2 | 5 |
| **Age** Average (min, max, n) | **60** (21-78, n = 44) | **60** (25-82, n = 45) | **60** (21-82, n = 89) |
| **Stage** | **50** | **54** | **104** |
| FIGO I-II | 9 | 2 | 11 |
| FIGO III-IV | 22 | 33 | 55 |
| unknown | 19 | 19 | 38 |

**Supplementary Table1: patients characteristics.**

**References**

1. Valous, N.A.*, et al.* Spatial intratumoral heterogeneity of proliferation in immunohistochemical images of solid tumors. *Med. Phys.* **43**, 2936-2947 (2016).

2. Ruifrok, A.C. & Johnston, D.A. Quantification of histochemical staining by color deconvolution. *Anal. Quant. Cytol. Histol.* **23**, 291-299 (2001).

3. Valous, N.A., Lahrmann, B., Zhou, W., Veltkamp, R. & Grabe, N. Multistage histopathological image segmentation of Iba1-stained murine microglias in a focal ischemia model: methodological workflow and expert validation. *J. Neurosci. Methods* **213**, 250-262 (2013).

4. Irlbeck, T.*, et al.* Association between single-slice measurements of visceral and abdominal subcutaneous adipose tissue with volumetric measurements: the Framingham Heart Study. *Int J Obes (Lond)* **34**, 781-787 (2010).

5. Nattenmueller, J.*, et al.* CT-based compartmental quantification of adipose tissue versus body metrics in colorectal cancer patients. *Eur Radiol* **26**, 4131-4140 (2016).

6. Yoshizumi, T.*, et al.* Abdominal fat: standardized technique for measurement at CT. *Radiology* **211**, 283-286 (1999).
